# Diverse reactivity of an iron–aluminium complex with substituted pyridines†

**DOI:** 10.1039/d2cc04498f

**Published:** 2022-09-02

**Authors:** Nikolaus Gorgas, Andrew J. P. White, Mark R. Crimmin

**Affiliations:** Department of Chemistry, Molecular Sciences Research Hub, Imperial College London, 82 Wood Lane, Shepherds Bush London W12 0BZ UK m.crimmin@imperial.ac.uk

## Abstract

The reaction of an Fe–Al complex with an array of substituted pyridines is reported. Depending on the substitution pattern of the substrate site-selective sp^2^ or sp^3^ C–H bond activation is observed. A series of reaction products are observed based on (i) C–Al bond formation, (ii) C–C bond formation by nucleophilic addition or (iii) deprotonation of the β-diketiminate ligand. A divergent set of mechanisms involving a common intermediate is proposed.

## Abstract 2

The reaction of an Fe–Al complex with an array of substituted pyridines is reported.

## Graphical abstract

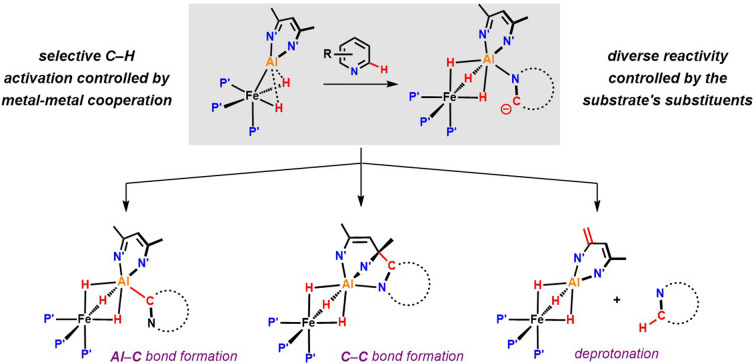

The transition metal mediated C–H activation and functionalisation of pyridines is a synthetic methodology of interest.^1^ In the last few years new strategies have emerged for the C–H activation of pyridines that are based on cooperative action of transition metal and main group elements contained within a single reactive species.^2–12^ Very recently, our group reported a well-defined Fe–Al complex that is capable of selectively breaking the sp^2^ C–H bond of pyridine (Fig. 1).^13^

**Fig. 1:**
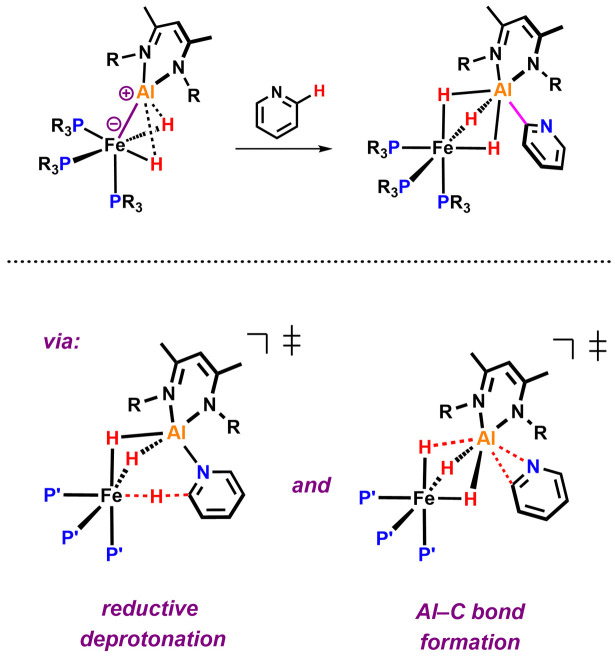
Elementary steps of the bimetallic *ortho* C–H activation of pyridine.

Based on a combination of kinetics experiments and DFT calculations, we proposed that C–H bond activation occurred *via* a novel mechanism involving two consecutive elementary steps. In the first step, the C–H bond is broken through a reductive deprotonation by the Fe centre. In a second step, the deprotonated pyridyl group rearranges to form a strong Al–C bond, a process that drives the thermochemistry of the reaction. During our efforts to expand the scope of this reaction to substituted pyridines, we observed a series of unexpected results. Modification of the pyridine revealed a series of divergent pathways in which the proposed deprotonated intermediate can attack electrophilic or acidic sites of the β-diketiminate ligand.^14^ These unforeseen reactions provide additional experimental support for the proposed mechanism of C–H activation through reductive deprotonation^13^ and highlight the non-innocence of the β-diketiminate ligand in this system.

We previously reported that the reaction of 1 with 4-methylpyridine in C_6_D_6_ resulted in the formation of 2 due to metalation of a sp^2^ C–H bond (Fig. 2). We can now conclude that, 2 is only the kinetic product of this reaction. Heating a C_6_D_6_ solution of 2 to 80 °C for 18 h, resulted in conversion into the thermodynamic product 3. 3 exhibits a sharp singlet resonance in the ^31^P{^1^H} NMR spectrum at *δ*_P_ = 29.0 ppm as well as a broadened quartet resonance at *δ*_H_ = −15.57 ppm in the ^1^H NMR spectrum. 3 clearly possesses a lower symmetry than 2 in solution. Most characteristic is the appearance of two distinct ^13^C{^1^H} resonances at *δ*_c_ = 145.5 and 62.0 ppm for the quaternary carbons of the β-diketiminate, indicative for the presence one sp^2^ and one sp^3^ C–N moiety, respectively. Compound 3 is proposed to arise from C- to N-rearrangement of the pyridyl anion followed by a nucleophilic attack on one of the imine positions of the β-diketiminate ligand (Fig. 2). Examples for this kind of reactivity are known but rare.^15–18^

**Fig. 2:**
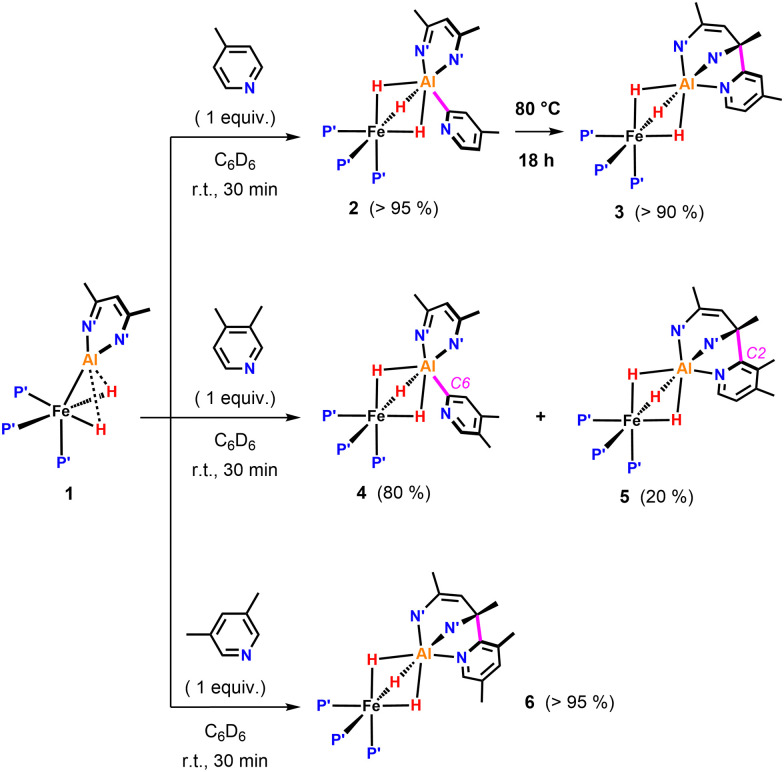
sp^2^ C–H activation of pyridines by 1. P′ = PMe_3_, N′ = N(2,4,6-MeC_6_H_2_).

Reaction of 1 with 3,4-dimethylpyridine under the same conditions gave a mixture of two products in a 4 : 1 ratio (Fig. 2). The major species 4 originated from C–H activation of the C^6^-position of the substrate. The minor species 5 most likely results from the C–H activation of the C^2^-position followed by attack on the β-diketiminate ligand.

The ratio of 4 and 5 does not change over time suggesting that these species are not in equilibrium. Similarly, reaction of 1 with 3,5-dimethylpyridine in C_6_D_6_ at 25 °C exclusively led to the formation of 6, the product of nucleophilic addition to the ligand (Fig. 2). Contrary to the observed site selectivity in the present case, the reaction of 4-methyl and 3,5-dimethylpyridine with the parent Al(i) complex [^Dipp^BDIAl]^19,20^ results in activation of remote CH or CH_3_ groups in the *para*-positions of these substrates.^21^ This provides a further example how heterobimetallic cooperativity can affect the reactivity or selectivity of a main group species.^22–24^

Further variation of the substrate revealed that 1 can react selectively at sp^3^ C–H bonds of pyridines in the presence of sp^2^ C–H bonds. Hence, addition of 2-methylpyridine to 1 resulted in deprotonation of the CH_3_ group to afford 7 in >95% NMR yield (Fig. 3). In the ^1^H NMR spectrum of 7, the new CH_2_ group exhibits diastereotopic protons characterised by two doublets at *δ*_H_ = 3.70 and 3.06 ppm with a mutual coupling constant of ^3^*J*_H–H_ = 17.4 Hz. The ^13^C{^1^H} NMR shows a resonance at *δ*_C_ = 59.4 ppm for the quaternary sp^3^ carbon created upon nucleophilic addition of the pyridyl group to the β-diketiminate ligand.^25^ Heating a C_6_D_6_ solution of 7 to 80 °C for 18 h leads to the formation of 8 in which the deprotonated sidearm of the 2-methylpyridine substrate is bound to the aluminium centre.

**Fig. 3:**
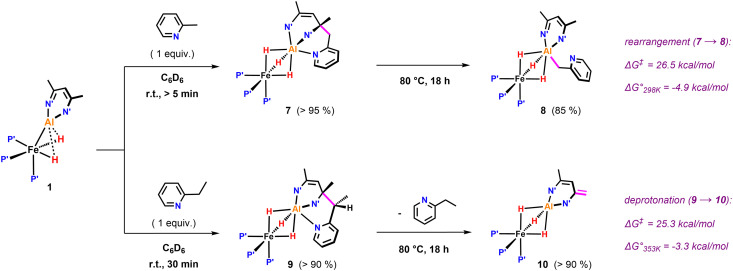
sp^3^ C–H activation of pyridines by 1: β nucleophilic attack at the ligand backbone at elevated temperatures. P′ = PMe_3_, N′ = N(2,4,6-MeC_6_H_2_).

Selective sp^3^ C–H bond activation was also observed upon the addition of 2-ethylpyridine to 1 which quantitatively formed 9. Heating a C_6_D_6_ solution of 9 to 80 °C for 18 h does not lead to the formation of the Al–C bound product, but rather reveals an alternative fate for the proposed anionic intermediate. The reaction results in the deprotonation^14,21,26^ of the methyl group of the β-diketiminate ligand and release of free 2-ethylpyridine. The diagnostic CH_2_ group in 10 gives rise to two doublets at *δ*_H_ = 3.88 and 3.16 ppm (^3^*J*_H–H_ = 1.4 Hz) in the ^1^H NMR spectrum. In the solid-state structure of 10 (Fig. 4), the CH_2_ and CH_3_ groups of the deprotonated ligand are disordered and appear averaged over positions related by a symmetry plan. The respective C–C bond distances are therefore almost equal (1.432(5) Å, 1.439(3) Å) but significantly shorter in comparison to those in the parent complex 1 (1.505(4) Å).

**Fig. 4:**
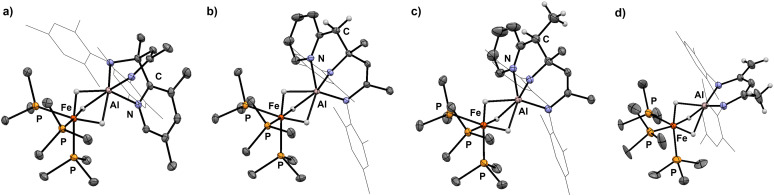
Crystal structures of 6 (a), 7 (b), 9 (c) and 10 (d).

DFT calculations were conducted to get a further insight into this divergent reactivity.^27^ 2-Methylpyridine was chosen as a suitable model substrate for these calculations as it allows consideration of both the site-selectivity of C–H activation and divergent fate of the proposed anionic intermediate.

Activation of a sp^3^ C–H at the methyl substituent was calculated to occur by reductive deprotonation mechanism, analogous to that previously reported for 1 (Fig. 5).^13^ The transition state for breaking the sp^3^ C–H bond (TS-1a) was found to be lower in energy (Δ*G*^‡^ = 13.7 *vs.* 20.8 kcal mol^−1^) than for the competing activation of the sp^2^ C–H bond in the 6-position (TS-1b). This finding is consistent with the role of the Fe site as a base in the mechanism and the known pKas of the sp^3^ and sp^2^ sites.^28–30^ The product of deprotonation, INT-2a is dearomatized and more stable than INT-2b, the corresponding intermediate from sp^2^ C–H activation (
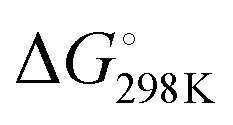
 = −4.2 *vs.* 16.1 kcal mol^−1^).

**Fig. 5:**
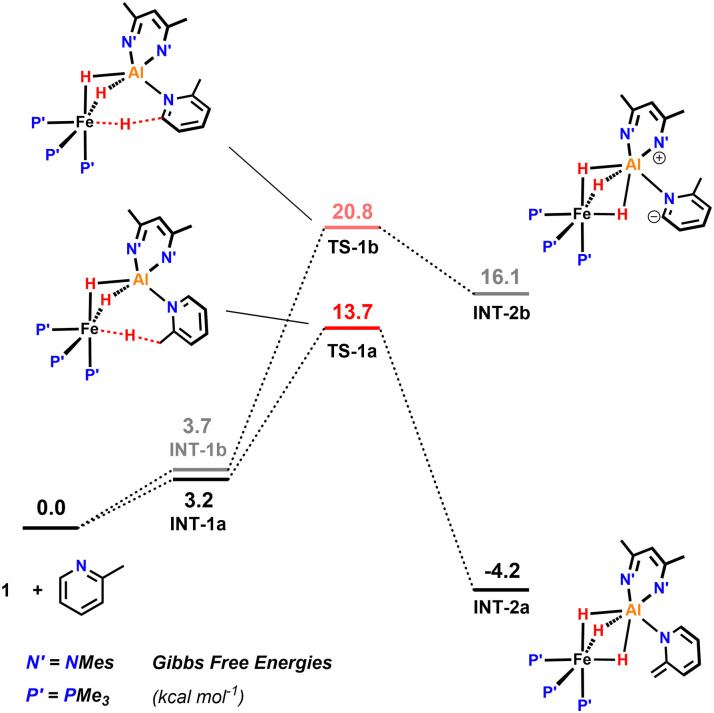
Calculations on the sp^3^ C–H activation of 2-methylpyridine in the reaction with 1 including the barrier for the competing activation of the sp^2^ C–H bond in the 4-position of the substrate.

Subsequent C–C bond formation from INT-2a (Fig. 6) is calculated to occur by rotation of the pyridyl around the Al–N bond fragment *via*TS-2 (Δ*G*^‡^ = 17.1 kcal mol^−1^) to form INT-3. Subsequent addition of the pyridyl group to the C=N position of the β-diketiminate ligand of INT-3 occurs *via*TS-3 (Δ*G*^‡^ = 11.7 kcal mol^−1^) to form the kinetic product 7. Calculations suggest that the key C–C bond forming step occurs by nucleophilic addition. The dearomatised intermediate INT-3 contains an enamide fragment. NBO calculations support the build on of charge on this fragment (N = −0.84; C = +0.18, CH_2_ = −0.60) that dissipates as C–C bond formation begins to occur in TS-3 (N = −0.71; C = +0.26, CH_2_ = −0.65). Similarly, the C=N position of the β-diketiminate backbone is electrophilic in INT-3 (C=N, +0.36) with charge accumulation occurring at this site during nucleophilic attack in TS-3 (C=N, +0.33). The pathway is reminiscent of classical aldol chemistry using enamide intermediates. The highest barrier in this sequence is still almost 4 kcal mol^−1^ lower than the activation energy required for the competing C–H activation of the sp^2^ C–H bond.

**Fig. 6:**
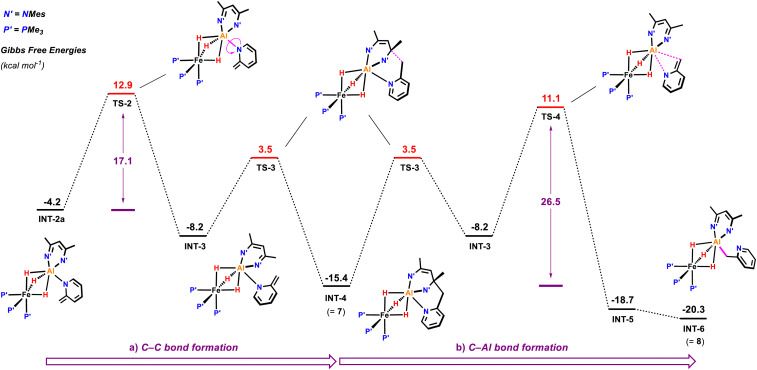
Calculated free energy profile for the rearrangement of the deprotonated 2-methypyridyl fragment in INT-2a. (a) Formation of the kinetic product 7 and (b) of the thermodynamic product 10. Gibbs free energies are given in kcal.mol^−1^ relative to 1+2-methylpyridine.

Conversion of 7 to the thermodynamic product 8, is calculated to occur through the microscopic reverse of the C–C bond forming step (*cf.* retro-aldol reaction) followed by an N- to C-pyridyl rearrangement of INT-3 to INT-5, through TS-4 (Δ*G*^‡^ = 26.5 kcal mol^−1^). This rearrangement involves a 1,3-sigmatropic shift and TS-4 resembles an aza-allyl intermediate. NBO calculations suggest a redistribution of charge in TS-4 (N = −0.69; C = +0.22, CH_2_ = −0.82) relative to that described for INT-3 above. 8 is calculated to be 4.9 kcal mol^−1^ lower in energy than 7.

Based on our current understanding, it is likely that the proposed mechanistic network is accessible for the entire series of substrates, but that differing substitution patterns on pyridine influence the barrier heights and relative thermodynamic stability of the products. For example, for 4-methypyridine the conversion of the Al–C product to the C–C product 2 → 3 is calculated to be exergonic by 
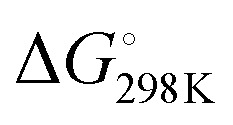
 = −7.1 kcal mol^−1^, the analogous reaction for 2-methylpyridine described above 8 → 7 is endergonic 
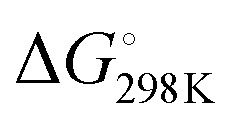
 = +4.9 kcal mol^−1^. The difference in thermochemistry is likely a consequence of the stability of the chelate (5 *vs.* 6 membered ring) formed in the C–C bond product, alongside the steric congestion of the Al–C product that occurs when the pyridyl group contains methyl substituents. Consistent with this latter argument, in no case are Al–C bonded products observed with an *ortho*-methyl substituent on pyridyl ring. Rather it appears this substitution pattern may destabilise the Al–C bonded product and favour the N- to C-rearrangement and C–C bond formation.

In summary, the reaction of an Fe–Al complex with several substituted pyridines has been reported. A series of divergent pathways have been identified that result in (i) C–Al bond formation, (ii) C–C bond formation, and (iii) ligand deprotonation. Common to these pathways is the formation of an anionic intermediate generated by site-selective deprotonation of either sp^2^ or sp^3^ positions of the pyridine substrate. The data provide additional support for a novel mechanism of C–H activation by heterometallic complexes previously reported by our group, they also highlight the potential non-innocence of the β-diketiminate ligand in these systems.

NG is grateful to the Austrian Science Fund (FWF) for provision of an Erwin Schrödinger Fellowship (Project No. J-4399).

## Conflicts of interest

There are no conflicts to declare.

## Supplementary Material

CC-058-D2CC04498F-s001

CC-058-D2CC04498F-s002

CC-058-D2CC04498F-s003

CC-058-D2CC04498F-s004

CC-058-D2CC04498F-s005

CC-058-D2CC04498F-s006
