# Supplementary material for: Diverse reactivity of an iron–aluminium complex with substituted pyridines
Source: Chem Commun (Camb). 2022 Sep 2;58(77):10849–52. doi: 10.1039/d2cc04498f (PMC9514013; doi:10.1039/d2cc04498f)
Supplement: CC-058-D2CC04498F-s005 [file CC-058-D2CC04498F-s005.pdf]

Electronic Supplementary Material (ESI) for ChemComm.
This journal is © The Royal Society of Chemistry 2022

Diverse Reactivity of an Iron–Aluminium Complex with Substituted Pyridines

*Nikolaus Gorgas, Andrew J. P. White, and Mark R. Crimmin**
Department of Chemistry, Imperial College London, White City, London, W12 0BZ, UK

1. General Experimental S2

2. Synthetic Procedures S3

3. X-Ray Data S11

4. Computational Methods S17

5. NMR Spectra of the isolated compounds S19

6. References S31

1. General Experimental

All manipulations were carried out using standard Schlenk-line and glovebox techniques under an inert atmosphere of argon or dinitrogen. A MBraun Labmaster glovebox was employed, operating at <0.1 ppm O_2 and <0.1 ppm H_2O. Solvents were dried over activated alumina from a SPS (solvent purification system) based upon the Grubbs design and degassed before use. Glassware was dried for 12 h at 120°C prior to use. C_6D_6 was dried over 3 Å molecular sieves and freeze-pump-thaw degassed thrice before use. Chemicals were purchased from Sigma Aldrich, Fluorochem, Alfa Aesar, and VWR. Pyridine substrates were dried over CaH_2, distilled, and stored over activated 3 Å molecular sieves. **1** and **2** were prepared as reported previously by our group.[1] NMR Spectra were recorded on Bruker 400 MHz or 500 MHz at 298 K unless otherwise stated and values recorded in ppm. Data were processed in MestReNova software. Where needed, chemical shifts were assigned with the assistance of 2D NMR (HSQC, HMBC, COSY) spectra. Elemental analyses were performed by Elemental Labs (https://www.elementallab.co.uk/).

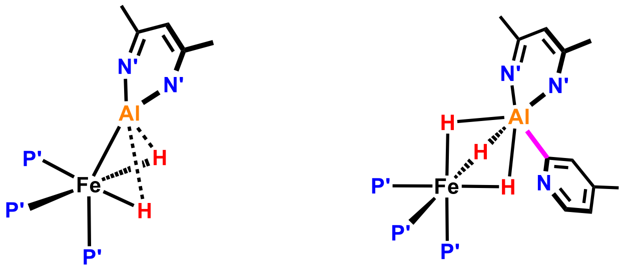

1 2

Figure S1. Line drawings of complexes 1 and 2. P' = PMe_3, N' = N(2,4,6-MeC_6H_2).

2. Synthetic Procedures

*Synthesis of **3**:*

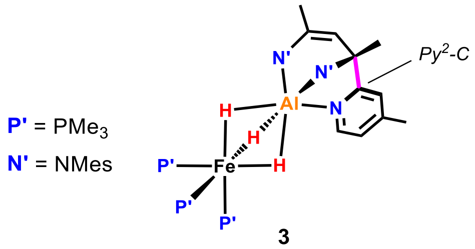

3

In a glovebox, **2** (15 mg, 0.020 mmol) was dissolved in C_6D_6 (0.6 mL) and transferred to a J-Young NMR tube. The NMR tube was sealed and heated to 80°C for 18 h in an isothermal bath during which time the reaction solution turns from pale yellow to orange. NMR analysis at the end of the reaction time revealed complete consumption of the **2** and formation of **3** in >90 % yield (based on integration of the ^{31}P resonances vs. PPh_3 as an internal standard). The reaction solution was transferred back to a glovebox and the solvent was removed under reduced pressure. The remaining sticky solid was triturated in *n*-pentane and the solvent again removed under reduced pressure. The resulting orange solid was washed with small amounts (3 x 0.5 ml) of *n*-pentane and dried under high vacuum. Isolated yield: 12.6 mg (0.017 mmol, 86 %).

^{1}H NMR (400 MHz, C_6D_6, 298 K): δ 8.44 (d, $^3J_{H–H}$ = 5.5 Hz, 1H, Py-C***H***), 7.13 (s, 1H, Mes-C***H***), 6.98 (s, 1H, Py-C***H***), 6.92 (s, 1H, Mes-C***H***), 6.89 (s, 1H, Mes-C***H***), 6.80 (s, 1H, Mes-C***H***), 6.51 (d, $^3J_{H–H}$ = 5.5 Hz, 1H, Py-C***H***), 4.26 (s, 1H, BDI-C***H***), 3.06 (s, 3H, Mes-CC***H***$_3$), 2.85 (s, 3H, Mes-CC***H***$_3$), 2.34 (s, 3H, Mes-CC***H***$_3$), 2.26 (s, 3H, Mes-CC***H***$_3$), 2.09 (s, 3H, Mes-CC***H***$_3$), 1.95 (s, 3H, Mes-CC***H***$_3$), 1.76 (s, 3H, Py^4-CC***H***$_3$), 1.49 (s, 3H, BDI-C(sp^2)C***H***$_3$), 1.45 (s, 3H, BDI-C(sp^3)C***H***$_3$), 0.97 – 0.91 (s, 27H, P-C***H***$_3$), -15.57 (q, $^2J_{P–H}$ = 21.0 Hz, 3H, Fe-***H***-Al).

^{31}P{^{1}H} NMR (162 MHz, C_6D_6, 298 K): δ 29.0 (s, 3P).

^{13}C{^{1}H} NMR (101 MHz, C_6D_6, 298 K): δ 168.7 (1C, Py^2-***C***), 150.2 (1C, Mes-***C***N), 149.2 (1C, Py^4-***C***CH$_3$), 148.8 (1C, Mes-***C***N), 145.5 (1C, BDI-***C***(sp^2)N), 141.8 (1C, Mes-***C***CH$_3$), 141.4 (1C, Py-***C***H), 139.9 (1C, Mes-***C***CH$_3$), 138.2 (1C, Mes-***C***CH$_3$), 138.0 (1C, Mes-***C***CH$_3$), 131.9 (1C, Mes-***C***CH$_3$), 130.1 (1C, Mes-***C***CH$_3$), 128.8 (1C, Mes-***C***H), 128.4 (1C, Mes-***C***H), 127.7 (1C, Mes-***C***H), 127.4 (1C, Mes-***C***H), 119.6 (1C, Py-***C***H), 116.6 (1C, Py-***C***H), 99.0 (1C, BDI-***C***H), 62.0 (1C, BDI-***C***(sp^3)N), 25.8 (m, 9C, P-***C***H$_3$), 24.0 (1C, BDI-C***C***H$_3$), 23.5 (1C, BDI-C***C***H$_3$), 22.0 (1C, Mes-C***C***H$_3$), 21.9 (1C, Mes-C***C***H$_3$), 21.0 – 20.7 (4C, 3 x Mes-C***C***H$_3$ + Py^4-C***C***H$_3$), 19.5 (1C, Mes-C***C***H$_3$).

Anal. Calc. ($C_{38}H_{65}AlFeN_3P_3$): C, 61.70; H, 8.86; N, 5.68. Found: C, 63.03; H, 8.74; N, 5.58.

*Synthesis of **4** and **5**:*

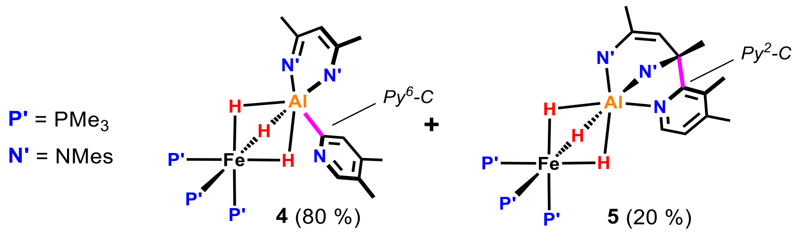

In a glovebox, a stock solution of 3,4-dimethylpyridine in C_6D_6 (0.1 M, 230 µL, 0.023 mmol) was added to a solution of **1** (15 mg, 0.023 mmol) in C_6D_6 (0.6 mL) and transferred to a J. Young NMR tube. Within 30 min, the colour of the reaction solution changed from red-orange to yellow. NMR analysis of the reaction solution revealed formation of **4** and **5** in a 4:1 ratio (>95 % NMR yield based on the relative integrals of in the ^{31}P NMR spectrum). The reaction solution was transferred back to a glovebox and the solvent was removed under reduced pressure. The remaining sticky solid was triturated in *n*-pentane and the solvent again removed under reduced pressure to afford an orange solid. Isolated yield: 15.6 mg (0.021 mmol, 90 %, **4**:**5** = 82:18).

*Spectroscopic data for **4**:*

^{1}H NMR (400 MHz, C_6D_6, 298 K): δ 8.81 (s, 1H, Py-C***H***), 7.94 (s, 1H, Py-C***H***), 6.79 (s, 2H, Mes-C***H***), 6.75 (s, 2H, Mes-C***H***), 5.27 (s, 1H, BDI-C***H***), 2.49 (s, 6H, Mes-CC***H***$_3$), 2.29 (s, 6H, Mes-CC***H***$_3$), 2.18 (s, 6H, Mes-CC***H***$_3$), 2.15 (s, 3H, Py-CC***H***$_3$), 2.04 (s, 3H, Py-CC***H***$_3$), 1.55 (s, 6H, BDI-CC***H***$_3$), 1.05 – 0.98 (m, 27H, P-C***H***$_3$), -15.32 (q, $^{2}J_{P\text{–}H}$ = 22.0 Hz, 3H, Fe-***H***-Al).

^{31}P{^{1}H} NMR (162 MHz, C_6D_6, 298 K): δ 29.7 (s, 3P).

^{13}C{^{1}H} NMR (101 MHz, C_6D_6, 298 K): δ 167.7 (2C, BDI-***C***N), 148.8 (1C, Py-***C***H), 146.4 (2C, Mes-***C***N), 137.7 (1C, Py-***C***CH$_3$), 136.1 (2C, Mes-***C***CH$_3$), 134.6 (1C, Py-***C***H), 133.7 (2C, Mes-***C***CH$_3$), 133.2 (2C, Mes-***C***CH$_3$), 129.8 (2C, Mes-***C***H), 129.0 (2C, Mes-***C***H), 126.4 (1C, Py-***C***CH$_3$), 100.4 (1C, BDI-***C***H), 25.8 (m, 9C, P-***C***H$_3$), 23.9 (2C, BDI-C***C***H$_3$), 21.3 (2C, Mes-C***C***H$_3$), 20.5 (2C, Mes-C***C***H$_3$), 20.2 (2C, Mes-C***C***H$_3$), 19.4 (1C, Py-C***C***H$_3$), 16.0 (1C, Py-C***C***H$_3$). The Py^{6}-***C***-Al resonance could not be observed due to line-broadening associated with coupling to the quadrupolar I = 5/2 ^{27}Al nucleus.

*Spectroscopic data for **5**:*

^{1}H NMR (400 MHz, C_6D_6, 298 K): δ 8.47 (d, $^{3}J_{H\text{–}H}$ = 5.4 Hz, 1H, Py-C***H***), 7.12 (s, 2H, Mes-C***H***), 6.91 (s, 1H, Mes-C***H***), 6.88 (s, 1H, Mes-C***H***), 6.60 (d, $^{3}J_{H\text{–}H}$ = 5.5 Hz, 1H, Py-C***H***), 4.59 (s, 1H, BDI-C***H***), 3.05 (s, 3H, C***H***$_3$), 2.85 (s, 3H, C***H***$_3$), 2.34 (s, 3H, C***H***$_3$), 2.24 (s, 3H, C***H***$_3$), 2.10 (s, 3H, C***H***$_3$), 2.04 (s, 3H, overlapped C***H***$_3$), 1.82 (s, 3H, C***H***$_3$), 1.77 (s, 3H, C***H***$_3$), 1.62 (s, 3H, C***H***$_3$), 1.51 (s, 3H, C***H***$_3$), 0.95 (s, 27H, P-C***H***$_3$), -15.57 (br, 3H, Fe-***H***-Al).

^{31}P{^{1}H} NMR (162 MHz, C_6D_6, 298 K): δ 28.9 (s, 3P).

The ^{13}C{^{1}H} NMR resonances of **5** could not be detected due to its low concentration in the product mixture.

*Synthesis of **6:***

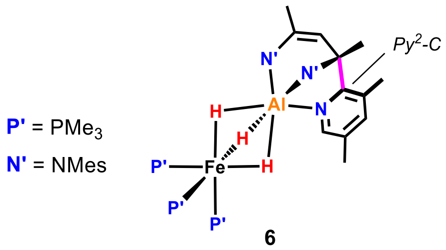

6

In a glovebox, a stock solution of 3,5-dimethylpyridine in C_6D_6 (0.1 M, 230 µL, 0.023 mmol) was added to a solution of **1** (15 mg, 0.023 mmol) in C_6D_6 (0.6 mL) and transferred to a J. Young NMR tube. Within 30 min, the colour of the reaction solution changed from red-orange to bright orange. NMR analysis of the reaction time revealed complete consumption of the **1** and formation of **6** in >95 % yield (based on the relative integrals of in the ^{31}P NMR spectrum). The reaction solution was transferred back to a glovebox and the solvent was removed under reduced pressure. The remaining sticky solid was triturated in *n*-pentane and the solvent again removed under reduced pressure. The resulting orange solid was dissolved in *n*-hexane (3 mL), the solution filtered and left at -35°C to form bright yellow crystals which were washed with cold *n*-pentane (3 x 0.5 mL) and dried under high vacuum. Isolated yield: 11.8 mg (0.016 mmol, 68 %). Crystals suitable for X-ray diffraction were obtained from a saturated *n*-hexane solution of **6** at -35°C.

^{1}H NMR (400 MHz, C_6D_6, 298 K): δ 8.42 (s, 1H, Py-C***H***), 7.12 (s, 1H, Mes-C***H***), 6.91 (s, 1H, Mes-C***H***), 6.87 (s, 1H, Mes-C***H***), 6.78 (s, 1H, Mes-C***H***), 6.74 (s, 1H, Py-C***H***), 4.54 (s, 1H, BDI-C***H***), 3.04 (s, 3H, Mes-C***H***3), 2.85 (s, 3H, Mes-C***H***3), 2.35 (s, 3H, Mes-C***H***3), 2.25 (s, 3H, Mes-C***H***3), 2.20 (s, 3H, Py-CC***H***3), 2.05 (s, 3H, dd), 2.00 (s, 3H, Py-CC***H***3), 1.78 (s, 3H, Mes-CC***H***3), 1.56 (s, 3H, BDI-C(sp^3)C***H***3), 1.49 (s, 3H, BDI-C(sp^2)C***H***3), 0.94 (br s, 27H, P-C***H***3), -15.60 (br, 3H, Fe-***H***-Al).

^{31}P{^{1}H} NMR (162 MHz, C_6D_6, 298 K): δ 28.9 (s, 3P).

^{13}C{^{1}H} NMR (101 MHz, C_6D_6, 298 K): δ 163.9 (1C, Py^2-***C***), 150.3 (1C, Mes-***C***N), 148.8 (1C, Mes-***C***N), 145.7 (1C, BDI-***C***(sp^2)N), 141.9 (1C, Mes-***C***CH3), 141.8 (1C, Py-***C***H), 139.8 (1C, Mes-***C***CH3), 139.6 (1C, Py-***C***H), 138.0 (1C, Mes-***C***CH3), 137.9 (1C, Mes-***C***CH3), 131.9 (1C, Mes-***C***CH3), 130.0 (1C, Mes-***C***CH3), 128.7 (1C, Mes-***C***H), 128.4 (1C, Mes-***C***H), 128.1 – 127.1 (3C, Py-***C***CH3 + 2 x Mes-***C***H) , 126.8 (1C, Py-***C***CH3), 96.2 (1C, BDI-***C***H), 64.0 (1C, BDI-***C***(sp^3)N), 25.7 (m, 10C, P-***C***H3 + BDI-C(sp^3)***C***H3), 23.6 (1C, BDI-C(sp^2)***C***H3), 22.0 – 21.9 (3C, Py-C***C***H3 + 2 x Mes-C***C***H3), 21.0 (1C, Mes-C***C***H3), 20.9 (1C, Mes-C***C***H3), 20.7 (1C, Mes-C***C***H3), 18.9 (1C, Mes-C***C***H3), 17.0 (1C, Py-C***C***H3).

Anal. Calc. ($C_{38}H_{65}AlFeN_3P_3$): C, 62.15; H, 8.96; N, 5.58. Found: C, 63.29; H, 8.70; N, 5.41.

*Synthesis of **7**:*

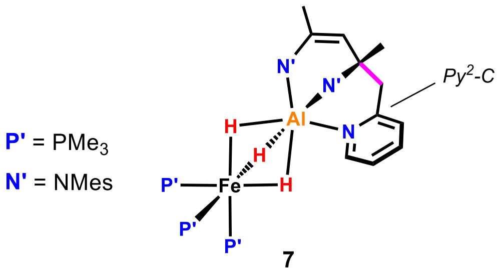

7

In a glovebox, a stock solution of 2-methylpyridine in C_6D_6 (0.1 M, 230 µL, 0.023 mmol) was added to a solution of **1** (15 mg, 0.023 mmol) in C_6D_6 (0.6 mL) and transferred to a J. Young NMR tube. The colour of the reaction solution immediately changed from red-orange to yellow. NMR analysis of the reaction time revealed complete consumption of the **1** and formation of **7** in >95 % yield (based on the relative integrals of in the ^{31}P NMR spectrum). The reaction solution was transferred back to a glovebox and the solvent was removed under reduced pressure. The remaining sticky solid was triturated in *n*-pentane and the solvent again removed under reduced pressure. The resulting orange solid was washed with small amounts (3 x 0.5 ml) of *n*-pentane and dried under high vacuum. Isolated yield: 15.8 mg (0.021 mmol, 93 %). Crystals suitable for X-ray diffraction were obtained by slow evaporation a saturated solution of **7** in *n*-hexane.

^{1}H NMR (400 MHz, C_6D_6, 298 K): δ 9.60 (d, $^{3}J_{H-H}$ = 5.2 Hz, 1H, Py-C***H***), 7.08 (s, 1H, Mes-C***H***), 6.98 (s, 1H, Mes-C***H***), 6.89 (apparent t, $^{3}J_{H-H}$ = 7.7 Hz, 1H, Py-C***H***), 6.86 (s, 1H, Mes-C***H***), 6.83 (s, 1H, Mes-C***H***), 6.65 (m, 2H, Py-C***H***), 4.38 (s, 1H, BDI-C***H***), 3.70 (d, $^{2}J_{H-H}$ = 17.4 Hz, 1H, Py^{2}-CC***H***$_2$), 3.06 (d, $^{2}J_{H-H}$ = 17.4 Hz, 1H, Py^{2}-CC***H***$_2$), 3.06 (s, 3H, Mes-CC***H***$_3$), 2.87 (s, 3H, Mes-CC***H***$_3$), 2.50 (s, 3H, Mes-CC***H***$_3$), 2.34 (s, 3H, Mes-CC***H***$_3$), 2.27 (s, 3H, Mes-CC***H***$_3$), 1.86 (s, 3H, Mes-CC***H***$_3$), 1.54 (s, 3H, BDI-CC***H***$_3$), 1.23 (s, 3H, BDI-CC***H***$_3$), 0.87 (s, 27H, P-C***H***$_3$), -15.79 (q, $^{2}J_{P-H}$ = 21.5 Hz, 3H, Fe-***H***-Al).

^{31}P{^{1}H} NMR (162 MHz, C_6D_6, 298 K): δ 28.2 (s, 3P).

^{13}C{^{1}H} NMR (101 MHz, C_6D_6, 298 K): δ 164.4 (1C, Py^{2}-***C***CH$_2$), 151.0 (1C, Mes-***C***N), 149.1 (1C, Py-***C***H), 149.0 (1C, Mes-***C***N), 143.3 (1C, BDI-***C***(sp^{2})N), 142.2 (1C, Mes-***C***CH$_3$), 139.1 (1C, Mes-***C***CH$_3$), 138.7 (1C, Mes-***C***CH$_3$), 138.0 (1C, Mes-***C***CH$_3$), 137.1 (1C, Py-***C***H), 131.7 (1C, Mes-***C***CH$_3$), 130.2 (1C, Mes-***C***CH$_3$), 128.9 (1C, Mes-***C***H), 128.6 (1C, Mes-***C***H), 128.6 (1C, Mes-***C***H), 128.5 (1C, Mes-***C***H), 126.8 (1C, Py-***C***H), 118.9 (1C, Py-***C***H), 103.7 (1C, BDI-***C***H), 59.4 (1C, BDI-***C***(sp^{3})N), 54.8 (1C, Py^{2}-C***C***H$_2$), 31.6 (1C, BDI-C***C***H$_3$), 25.5 (m, 9C, P-***C***H$_3$), 24.8 (1C, BDI-C***C***H$_3$), 23.1 (1C, Mes-C***C***H$_3$), 22.4 (1C, Mes-C***C***H$_3$), 21.7 (1C, Mes-C***C***H$_3$), 20.8 (1C, Mes-C***C***H$_3$), 20.6 (1C, Mes-C***C***H$_3$), 19.3 (1C, Mes-C***C***H$_3$).

Anal. Calc. ($C_{38}H_{65}AlFeN_3P_3$): C, 61.70; H, 8.86; N, 5.68. Found: C, 61.29; H, 8.36; N, 5.41.

Synthesis of ***8:***

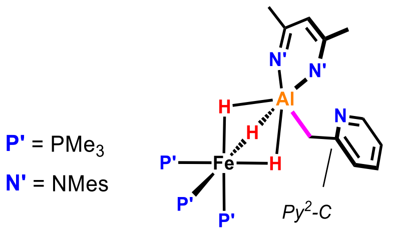

8

In a glovebox, **7** (20 mg, 0.031 mmol) was dissolved in C_6D_6 (0.6 mL) and transferred to a J-Young NMR tube. The NMR tube was sealed and heated to 80°C for 18 h in an isothermal bath during which time the reaction solution turns from pale yellow to orange. NMR analysis at the end of the reaction time revealed complete consumption of the **7** and formation of **8** in ca. 85 % yield (based on integration of the ^{31}P resonances vs. PPh_3 as an internal standard). The reaction solution was transferred back to a glovebox and the solvent was removed under reduced pressure. The remaining orange solid was washed with of *n*-pentane (3 x 1.0 ml) and dried under high vacuum. Isolated yield: 13.0 mg (0.017 mmol, 55 %).

^{1}H NMR (400 MHz, C_6D_6, 298 K): δ 8.43 (d, $^{3}J_{H\text{–}H}$ = 5.4 Hz, 1H, Py-C***H***), 7.17 (d, $^{3}J_{H\text{–}H}$ = 6.9 Hz, 1H, Py-C***H***), 7.11 (m, 1H, Py-C***H***), 6.85 (s, 2H, Mes-C***H***), 6.77 (s, 2H, Mes-C***H***), 6.61 (m, 1H, Py-C***H***), 4.61 (s, 1H, BDI-C***H***), 2.91 (s, 6H, Mes-CC***H***$_3$), 2.63 (s, 2H, Py^2-CC***H***$_2$), 2.32 (s, 6H, Mes-CC***H***$_3$), 2.19 (s, 6H, Mes-CC***H***$_3$), 1.43 (s, 6H, BDI-CC***H***$_3$), 0.94 (m, 27H, P-C***H***$_3$), -15.66 (bs, 3H).

^{31}P{^{1}H} NMR (162 MHz, C_6D_6, 298 K): δ 28.3 (s, 3P).

^{13}C{^{1}H} NMR (101 MHz, C_6D_6, 298 K): δ 173.4 (1C, Py^2-***C***CH$_2$), 168.1 (2C, BDI-***C***N), 147.9 (1C, Py-C***H***), 146.0 (1C, Mes-***C***N), 136.1 (2C, Mes-***C***CH$_3$), 133.8 (2C, Mes-***C***CH$_3$), 133.7 (2C, Mes-***C***CH$_3$), 133.3 (1C, Py-C***H***), 129.9 (2C, Mes-C***H***), 129.0 (2C, Mes-C***H***), 120.6 (1C, Py-C***H***), 114.4 (1C, Py-C***H***), 100.5 (1C, BDI-C***H***), 42.5 (located from the ^{1}H/^{13}C HSQC spectrum, 1C, Py^2-C***C***H$_2$), 26.2 (m, 9C, P-***C***H$_3$), 23.9 (2C, BDI-C***C***H$_3$), 21.5 (2C, Mes-C***C***H$_3$), 20.5 (2C, Mes-C***C***H$_3$), 20.0 (2C, Mes-C***C***H$_3$).

Anal. Calc. ($C_{38}H_{65}AlFeN_3P_3$): C, 61.70; H, 8.86; N, 5.68. Found: C, 60.40; H, 8.10; N, 5.44.

Synthesis of ***9:***

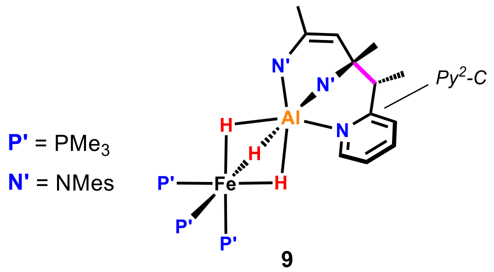

9

In a glovebox, a stock solution of 2-ethylpyridine in C_6D_6 (0.1 M, 230 µL, 0.023 mmol) was added to a solution of **1** (15 mg, 0.023 mmol) in C_6D_6 (0.6 mL) and transferred to a J. Young NMR tube. Within 30 min, the colour of the reaction solution changed from red-orange to bright orange. NMR analysis at the end of the reaction time revealed complete consumption of the **1** and formation of **9** in >90 % yield (based on the relative integrals of in the ^{31}P NMR spectrum). The reaction solution was transferred back to a glovebox and the solvent was removed under reduced pressure. The remaining sticky solid was triturated in *n*-pentane and the solvent again removed under reduced pressure. The resulting solid was washed with small amounts (3 x 0.5 ml) of *n*-pentane and dried under high vacuum. Isolated yield: 15.4 mg (0.020 mmol, 91 %). Crystals suitable for X-ray diffraction were obtained by slow evaporation a saturated solution of **9** in *n*-hexane.

^{1}H NMR (400 MHz, C_6D_6, 298 K): δ 9.61 (d, $^3J_{H-H}$ = 5.8 Hz, 1H, Py-C***H***), 7.06 (s, 1H, Mes-C***H***), 6.99 (m, 2H, Py-C***H***), 6.96 (s, 1H, Mes-C***H***), 6.88 (s, 2H, Mes-C***H***), 6.57 (apparent t, $^3J_{H-H}$ = 6.2 Hz, 1H, Py-C***H***), 4.24 (s, 1H, BDI-C***H***), 3.60 (q, $^3J_{H-H}$ = 7.1 Hz, 1H, Py-CC***H***CH$_3$), 3.08 (s, 3H, Mes-CC***H***$_3$), 2.89 (s, 3H, Mes-CC***H***$_3$), 2.33 (s, 3H, Mes-CC***H***$_3$), 2.31 (s, 3H, Mes-CC***H***$_3$), 2.28 (s, 3H, Mes-CC***H***$_3$), 2.09 (s, 3H, Mes-CC***H***$_3$), 1.65 (s, 3H, BDI-CC***H***$_3$), 1.39 (d, $^3J_{H-H}$ = 7.1 Hz, 3H, Py-CCHC***H***$_3$), 1.26 (s, 3H, BDI-CC***H***$_3$), 0.82 (m, 27H, P-C***H***$_3$), -15.90 (q, $^2J_{P-H}$ = 22.3 Hz, 3H, Fe-***H***-Al).

^{31}P{^{1}H} NMR (162 MHz, C_6D_6, 298 K): δ 28.0 (s, 3P).

^{13}C{^{1}H} NMR (101 MHz, C_6D_6, 298 K): δ 170.0 (1C, Py^2-***C***CHCH$_3$), 151.3 (1C, Mes-***C***N), 149.8 (1C, Py-***C***H), 149.1 (1C, Mes-***C***N), 142.7 (1C, BDI-***C***(sp^2)N), 142.3 (1C, Mes-***C***CH$_3$), 139.7 (1C, Mes-***C***CH$_3$), 138.6 (1C, Mes-***C***CH$_3$), 138.1 (1C, Mes-***C***CH$_3$), 137.2 (1C, Py-***C***H), 131.7 (1C, Mes-***C***CH$_3$), 130.2 (1C, Mes-***C***CH$_3$), 129.0 (2C, Mes-***C***H), 128.6 (1C, Mes-***C***H), 128.5 (1C, Mes-***C***H), 126.0 (1C, Py-***C***H), 118.1 (1C, Py-***C***H), 98.8 (1C, BDI-***C***H), 61.5 (1C, BDI-***C***(sp^3)N), 55.7 (1C, Py^2-C***C***HCH$_3$), 29.9 (1C, BDI-C***C***H$_3$), 25.5 (m, 9C, P-***C***H$_3$), 24.9 (1C, BDI-C***C***H$_3$), 23.2 (1C, Mes-C***C***H$_3$), 22.4 (1C, Mes-C***C***H$_3$), 21.7 (1C, Py^2-CCH***C***H$_3$), 21.5 (1C, Mes-C***C***H$_3$), 20.8 (1C, Mes-C***C***H$_3$), 20.7 (1C, Mes-C***C***H$_3$), 19.6 (1C, Mes-C***C***H$_3$).

Anal. Calc. ($C_{38}H_{65}AlFeN_3P_3$): C, 62.15; H, 8.96; N, 5.58. Found: C, 61.70; H, 8.86; N, 5.52.

Synthesis of ***10:***

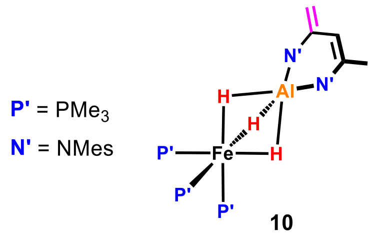

10

In a glovebox, **9** (20 mg, 0.027 mmol) was dissolved in C_6D_6 (0.6 mL) and transferred to a J-Young NMR tube. The NMR tube was sealed and heated to 80°C for 18 h in an isothermal bath. NMR analysis at the end of the reaction time revealed complete consumption of the **9** and formation of **10** in >90 % yield (based on integration of the ^{31}P resonances vs. PPh_3 as an internal standard). The reaction solution was transferred back to a glovebox and the solvent was removed under reduced pressure. The resulting solid was washed with small amounts (4 x 0.5 ml) of *n*-pentane and dried under high vacuum. Isolated yield: 12.8 mg (0.020 mmol, 76 %). Crystals suitable for X-ray diffraction were obtained by slow evaporation a saturated solution of **10** in diethyl ether.

^{1}H NMR (400 MHz, C_6D_6, 298 K): δ 6.97 (s, 2H, Mes-C***H***), 6.81 (s, 2H, Mes-C***H***), 5.56 (s, 1H, BDI-C***H***), 3.88 (s, 1H, BDI-CC***H***$_2$), 3.16 (s, 1H, BDI-CC***H***$_2$), 2.66 (s, 6H, Mes-CC***H***$_3$), 2.44 (s, 6H, Mes-CC***H***$_3$), 2.29 (s, 3H, Mes-CC***H***$_3$), 2.22 (s, 3H, Mes-CC***H***$_3$), 1.55 (s, 3H, BDI-CC***H***$_3$), 0.71 (m, 27H, P-C***H***$_3$), -16.06 (q, $^2J_{P\text{–}H}$ = 21.6 Hz, 3H, Fe-***H***-Al).

^{31}P{^{1}H} NMR (162 MHz, C_6D_6, 298 K): δ 27.6 (s, 3P).

^{13}C{^{1}H} NMR (101 MHz, C_6D_6, 298 K): δ 154.0 (1C, BDI-***C***(CH$_2$)N), 145.5 (1C, Mes-**C**N), 145.3 (1C, Mes-**C**N), 143.4 (1C, BDI-***C***(CH$_3$)N), 137.4 (2C, Mes-**C**CH$_3$), 137.2 (2C, Mes-**C**CH$_3$), 133.2 (1C, Mes-**C**CH$_3$), 132.9 (1C, Mes-**C**CH$_3$), 129.6 (2C, Mes-**C**H), 128.8 (2C, Mes-**C**H), 103.0 (1C, BDI-**C**H), 77.1 (1C, BDI-C**C**H$_2$), 25.3 (m, 9C, P-**C**H$_3$), 23.4 (1C, BDI-C**C**H$_3$), 20.8 (1C, Mes-C**C**H$_3$), 20.7 (1C, Mes-C**C**H$_3$), 19.8 (2C, Mes-C**C**H$_3$), 19.3 (2C, Mes-C**C**H$_3$).

Anal. Calc. ($C_{32}H_{58}AlFeN_2P_3$): C, 59.44; H, 9.04; N, 4.33. Found: C, 60.98; H, 8.26; N, 4.26.

*Synthesis of **S1**:*

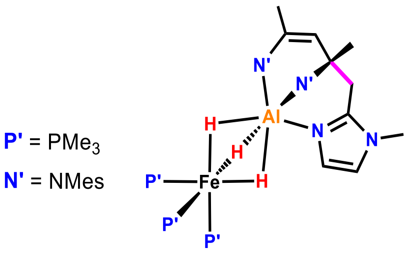

In a glovebox, a stock solution of 1,2-dimethylimidazole in C_6D_6 (0.1 M, 230 µL, 0.023 mmol) was added to a solution of **1** (15 mg, 0.023 mmol) in C_6D_6 (0.6 mL) and transferred to a J. Young NMR tube. The colour of the reaction solution immediately changed from red-orange to yellow. NMR analysis of the reaction time revealed complete consumption of the **1** and formation of **S1** in >95 % yield. The reaction solution was transferred back to a glovebox and the solvent was removed under reduced pressure. The remaining sticky solid was triturated in *n*-pentane and the solvent again removed under reduced pressure. The resulting orange solid was washed with small amounts (3 x 0.5 ml) of *n*-pentane and dried under high vacuum. Isolated yield: 16.4 mg (0.022 mmol, 96 %). Crystals suitable for X-ray diffraction were obtained by slow evaporation a saturated solution of **S1** in diethyl ether.

^{1}H NMR (400 MHz, C_6D_6, 298 K): δ 7.44 (s, 1H, Imidazole-C***H***), 7.11 (s, 1H, Mes-C***H***), 7.01 (s, 1H, Mes-C***H***), 6.91 (s, 1H, Mes-C***H***), 6.89 (s, 1H, Mes-C***H***), 6.01 (s, 1H, Imidazole-C***H***), 4.27 (s, 1H, BDI-C***H***), 3.10 (s, 3H, Mes-C***H***$_3$), 2.90 (s, 3H, Mes-C***H***$_3$), 2.89 (d, *J* = 16.1 Hz, 1H, Imidazole-C***H***$_2$), 2.51 (d, *J* = 16.1 Hz, 1H, Imidazole-C***H***$_2$), 2.45 (s, 3H, Mes-C***H***$_3$), 2.36 (s, 3H, Mes-C***H***$_3$), 2.30 (s, 3H, Mes-C***H***$_3$), 2.21 (s, 6H, Mes-C***H***$_3$ + Imidazole-NC***H***$_3$), 1.60 (s, 3H, BDI-C***H***$_3$), 1.30 (s, 3H, BDI-C***H***$_3$), 0.95 (s, 27H, P-C***H***$_3$), -15.71 (br q, *J* = 21.0 Hz, 3H, Fe-***H***-Al).

^{31}P{^{1}H} NMR (162 MHz, C_6D_6, 298 K): δ 28.8 (s, 3P).

^{13}C{^{1}H} NMR (101 MHz, C_6D_6, 298 K): δ 151.9 (1C, Mes-**C**N), 150.7 (1C, Imidazole-**C**CH$_2$), 149.6 (1C, Mes-**C**N), 143.9 (1C, BDI-**C**N), 142.4 (1C, Mes-**C**CH$_3$), 139.1 (1C, Mes-**C**CH$_3$), 138.6 (1C, Mes-**C**CH$_3$), 138.3 (1C, Mes-**C**CH$_3$), 131.4 (1C, Mes-**C**CH$_3$), 130.0 (1C, Mes-**C**CH$_3$), 128.7 (1C, Mes-**C**H), 128.5 (1C, Mes-**C**H), 128.4 (1C, Mes-**C**H), 128.4 (1C, Mes-**C**H), 126.0 (1C, Imidazole-**C**H), 117.0 (1C, Imidazole-**C**H), 102.5 (1C, BDI-**C**H), 59.8 (1C, BDI-**C**(sp^3)N), 43.9 (1C, Imidazole-C**C**H$_2$), 31.8 (1C, BDI-**C**H$_3$), 31.3 (1C, Imidazole-N**C**H$_3$), 25.9 (m, 9C, P-**C**H$_3$), 25.1 (1C, BDI-**C**H$_3$), 22.8 (1C, Mes-C**C**H$_3$), 22.0 (1C, Mes-C**C**H$_3$), 21.7 (1C, Mes-C**C**H$_3$), 20.9 (1C, Mes-C**C**H$_3$), 20.7 (1C, Mes-C**C**H$_3$), 20.1 (1C, Mes-C**C**H$_3$).

Anal. Calc. ($C_{37}H_{66}AlFeN_4P_3$): C, 59.84; H, 8.96; N, 7.54. Found: C, 59.85; H, 8.69; N, 7.54.

3. X-Ray Data

Table S1 provides a summary of the crystallographic data for the structures of **6**, **7**, **8**, **9**, **10** and **S1**. Data were collected using an Agilent Xcalibur PX Ultra A diffractometer, and the structures were solved and refined using the OLEX2,[2] SHELXTL[3] and SHELX-2013[4] program systems. CCDC 2195829 to 2195834.

Table S1. Crystal Data, Data Collection and Refinement Parameters for the structures of **6**, **7**, **8**, **9**, **10** and **S1**.

data	**6**	**7**	**8**
**formula**	$C_{39}H_{67}AlFeN_3P_3$	$C_{38}H_{65}AlFeN_3P_3$	$C_{38}H_{65}AlFeN_3P_3$
**solvent**	—	—	—
**formula weight**	753.69	739.67	739.67
**colour, habit**	yellow plates	orange tablets	pale yellow platy needles
**temperature / K**	173	173	173
**crystal system**	monoclinic	monoclinic	orthorhombic
**space group**	$P2_1/c$ (no. 14)	$P2_1/c$ (no. 14)	*Ibam* (no. 72)
***a* / Å**	14.6040(2)	12.84139(14)	16.6412(4)
***b* / Å**	16.8168(2)	18.42462(19)	30.2391(8)
***c* / Å**	17.4073(2)	17.8629(2)	16.6540(8)
**α / deg**	90	90	90
**β / deg**	99.8760(12)	102.3236(11)	90
**γ / deg**	90	90	90
**V / Å^3**	4211.74(10)	4128.94(8)	8380.5(5)
Z	4	4	8 [c]
**D_c / g cm^{-3}**	1.189	1.190	1.172
**radiation used**	Cu-Kα	Cu-Kα	Cu-Kα
**μ / mm^{-1}**	4.362	4.441	4.376
**no. of unique reflns**			
**measured (R_{int})**	8033 (0.0357)	7892 (0.0316)	3431 (0.0535)
**obs, $\|F_o\| > 4\sigma(\|F_o\|)$**	6502	6771	2454
**completeness (%)** [a]	98.4	98.1	81.0
**no. of variables**	455	445	268
**R_1(obs), wR_2(all)** [b]	0.0385, 0.0968	0.0350, 0.0933	0.0511, 0.01376

[a] Completeness to 0.84 Å resolution. [b] $R_1 = \Sigma||F_o| - |F_c||/\Sigma|F_o|$; $wR_2 = \{\Sigma[w(F_o^2 - F_c^2)^2] / \Sigma[w(F_o^2)^2]\}^{1/2}$; $w^{-1} = \sigma^2(F_o^2) + (aP)^2 + bP$. [c] The complex has crystallographic C_S symmetry.

Table S1. part 2

data	9	10	S1
**formula**	$C_{39}H_{67}AlFeN_3P_3$	$C_{32}H_{58}AlFeN_2P_3$	$C_{37}H_{66}AlFeN_4P_3$
**solvent**	—	—	0.5($C_4H_{10}O$)
**formula weight**	753.69	646.54	779.73
**colour, habit**	yellow tablets	pale yellow tablets	colourless plates
**temperature / K**	173	173	173
**crystal system**	monoclinic	monoclinic	monoclinic
**space group**	$P2_1/c$ (no. 14)	$I2/a$ (no. 15)	$P2_1/c$ (no. 14)
a **/ Å**	12.86606(17)	20.9505(4)	22.1904(4)
b **/ Å**	18.3430(2)	15.4700(3)	11.6107(2)
c **/ Å**	18.3536(3)	22.5791(4)	17.3595(3)
**α / deg**	90	90	90
**β / deg**	103.0614(14)	92.0326(15)	103.656(2)
**γ / deg**	90	90	90
V **/ Å³**	4219.44(10)	7313.4(2)	4346.20(15)
Z	4	8	4
D_c **/ g cm⁻³**	1.186	1.174	1.192
**radiation used**	Cu-Kα	Cu-Kα	Cu-Kα
**μ / mm⁻¹**	4.354	4.936	4.257
**no. of unique reflns**			
**measured (**R_{int}**)**	8079 (0.0365)	6957 (0.0288)	16408 (0.0919)
**obs,** $\|F_o\| > 4\sigma(\|F_o\|)$	6651	5723	9959
**completeness (%)** [a]	98.4	98.1	98.1
**no. of variables**	454	455	487
R_1**(obs),** wR_2**(all)** [b]	0.0460, 0.1265	0.0487, 0.1290	0.0623, 0.1749

X-ray crystallography

The three Al–H–Fe bridging hydrogen atoms in the structures of **6**, **7**, **9**, **10** and **S1**, and the two unique Al–H–Fe bridging hydrogen atoms in the structure of **8** were all located from Δ*F* maps and refined freely.

The crystal of **8** that was studied was a noticeably weak scatter of X-rays, and so the data collection was designed with low target intensities for both the low and high angle images in order to give a reasonable data collection time. Similarly, the data collection was also designed to only collect unique data, but unfortunately, the close similarity of the *a* and *c* axis lengths [16.6412(4) and 16.6540(8) Å respectively, though they appeared closer at the time the experiment was being designed] led to the presumption that the correct

crystal system was tetragonal, and so 4/*m* data was collected. Sometime after the crystal had already been removed from the diffractometer (and thus decomposed), but before the structure had been successfully solved, it was discovered that the correct crystal system was orthorhombic, making the data set incomplete (*ca*. 81% completeness to a resolution of 0.84 Å). Unfortunately, again, by this time the rest of the sample had also decomposed, making the incomplete data set all that was going to be collected. However, despite the incompleteness of the data set making the derived structure of inevitably lower quality than would otherwise be the case, there is still plenty of worthwhile information that can be gleaned from it. The structure of **8** was found to sit across a mirror plane that passes through C2, Al1 and Fe1, and bisects the N1···N1A vector. The C13-bound pyridyl ring and the methyl groups of the P20-based PMe_3 moiety were both found to be disordered across this mirror plane, and in each case one complete 50% occupancy orientation was identified (with a second 50% occupancy orientation being generated by operation of the mirror plane). The geometries of the two unique orientations were optimised, and all of the non-hydrogen atoms were refined anisotropically. The P24-based PMe_3 group was also found to be disordered. Two orientations were identified of *ca*. 54 and 46% occupancy, their geometries were optimised, the thermal parameters of adjacent atoms were restrained to be similar, and only the non-hydrogen atoms of the major occupancy orientation were refined anisotropically (those of the minor occupancy orientation were refined isotropically). Despite all the disorder and the incomplete data, the basic identity of the compound and its connectivity are clear, especially when considered with the other structures in this paper. The main effect of the incomplete data set is likely to be a degradation of the standard uncertainties, and this can be seen by a comparison of the Al···Fe separations across all six structures – the "well behaved" structures of **6**, **7**, **9** and **10** have su's of 0.0007, 0.0006, 0.0008 and 0.0008 Å respectively for this distance, whilst the structure of **S1** (with the twinned data set) has an s.u. of 0.0013 Å and the structure of **8** (with the incomplete data set) has an s.u. of 0.0015 Å.

The backbone of the deprotonated β-diketiminate ligand in the structure of **10** was found to be disordered, with the location of the terminal C–Me and C=CH_2 units being "swapped" between the C1/C4 and C3/C5 sites. This was modelled by using two sets of idealised partial-occupancy hydrogen atoms in a *ca*. 51:49 ratio — the carbon atoms were unaffected, and the occupancies of the hydrogen atoms were allowed to vary with no restraints other than the members of each set having to have the same occupancy as each other, and the total occupancy being 100%. The $Fe(PMe_3)_3$ moiety was also found to be disordered, and two orientations with a common iron atom position were identified of *ca*. 54 and 46% occupancy. The geometries of the two orientations were optimised, the thermal parameters of adjacent atoms were restrained to be similar, and only the non-hydrogen atoms of the major occupancy orientation, and the phosphorus centres of the minor occupancy orientation, were refined anisotropically (the rest were refined isotropically).

The crystal of **S1** that was studied was found to be a two component twin in a *ca*. 71:29 ratio, with the two lattices related by the approximate twin law [0.99 0.00 –0.02 0.01 1.00 0.02 0.03 –0.06 1.01]. The O50-based included diethylether solvent molecule was found to be disordered across a centre of symmetry, and two

unique orientations were identified of *ca*. 30 and 20% occupancy (with two further orientations of the same occupancies being generated by operation of the inversion centre). The geometries of the two unique orientations were optimised, the thermal parameters of adjacent atoms were restrained to be similar, and all of the atoms of both unique orientations were refined isotropically.

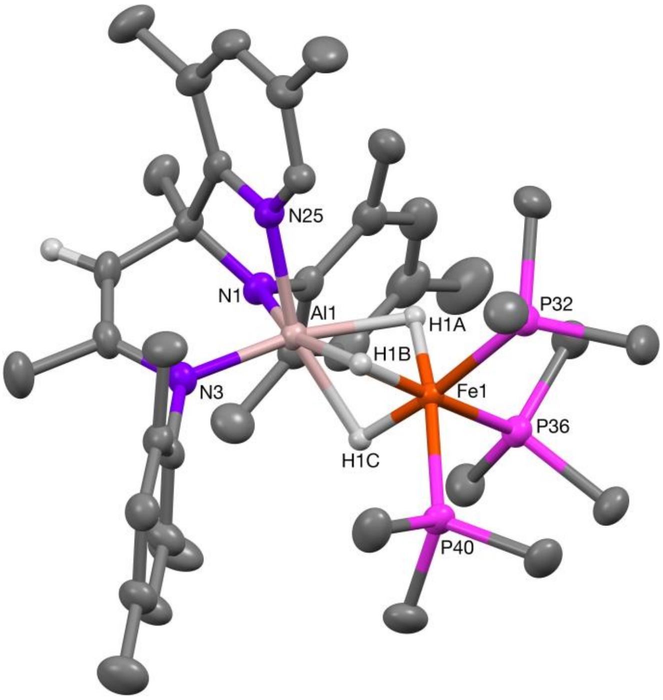

Figure S2. The crystal structure of **6** (50% probability ellipsoids).

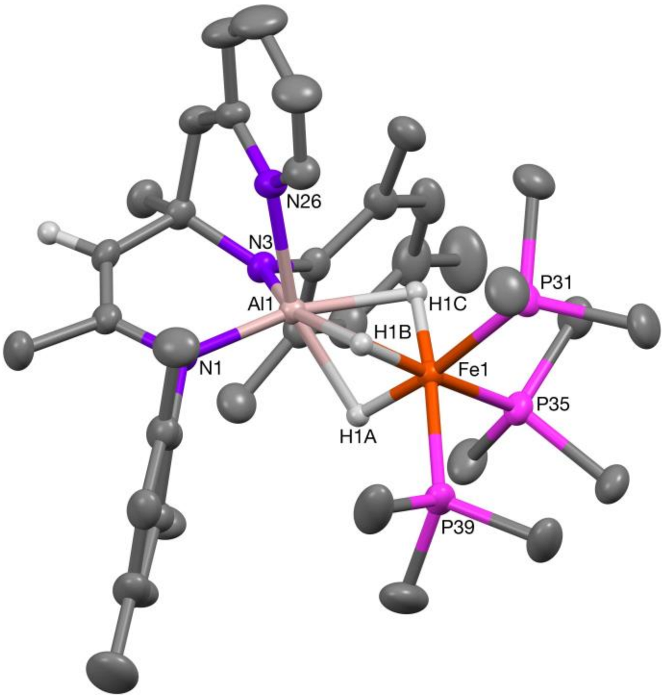

Figure S3. The crystal structure of **7** (50% probability ellipsoids).

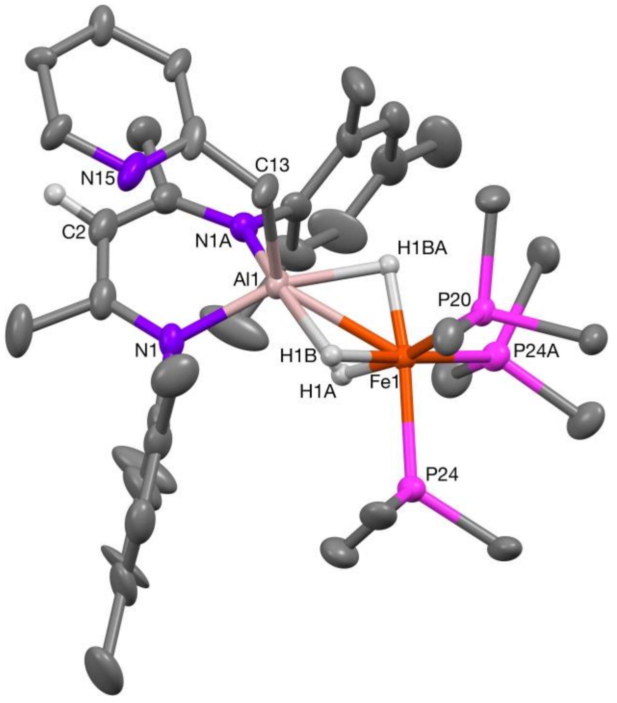

Figure S4. The crystal structure of the C_S-symmetric complex **8** (30% probability ellipsoids).

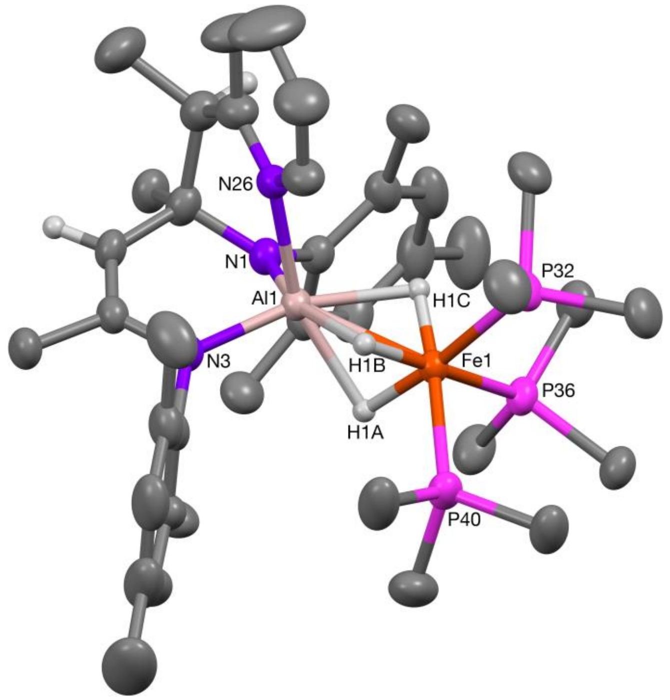

Figure S5. The crystal structure of **9** (50% probability ellipsoids).

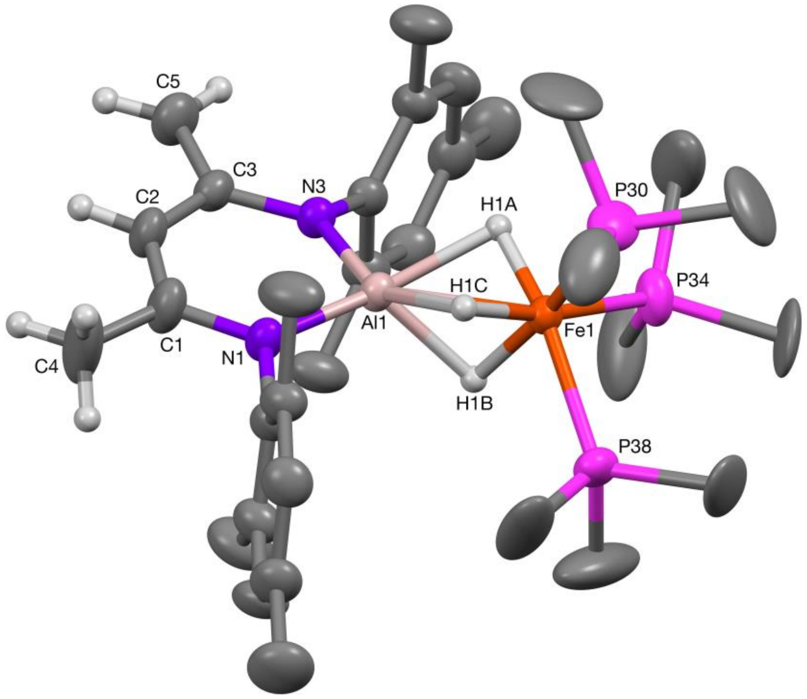

Figure S6. The crystal structure of **10** (50% probability ellipsoids).

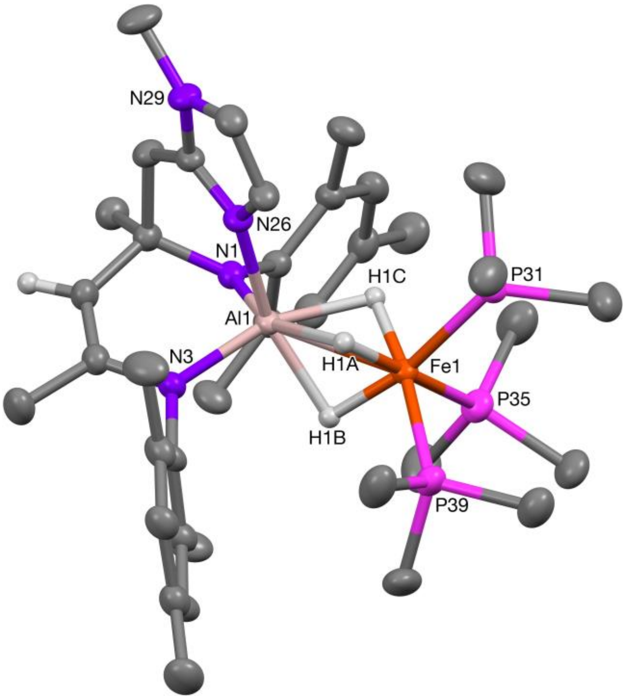

Figure S7. The crystal structure of **S1** (50% probability ellipsoids).

4. Computational Methods

DFT calculations were run using Gaussian 09 (Revision D.01).[5] Geometry optimisation calculations were performed without symmetry constraints. Frequency analyses for all stationary points were performed using the enhanced criteria to confirm the nature of the structures as either minima (no imaginary frequency) or transition states (only one imaginary frequency). Intrinsic reaction coordinate (IRC) calculations followed by full geometry optimisations on final points were used to connect transition states and minima located on the potential energy surface allowing a full energy profile (calculated at 298.15 K, 1 atm) of the reaction to be constructed.[6,7] Solvent corrections were applied using the polarizable continuum model (PCM).[8] Dispersion corrections were applied using Grimme's D3 (GD3) correction.[9] All calculations were conducted using the B3PW91[10] functional including solvent and dispersion corrections directly in the optimisations. Al and Fe centres were described with Stuttgart SDDAll ECP and associated basis sets, and the 6-31G** basis sets were used for all other atoms.[11,12,13] The employed level of theory has been benchmarked against experimental data as reported previously by our group.[1] Natural Bond Orbital analysis was carried out using NBO 6.0.[14]

Calculated thermodynamical data:

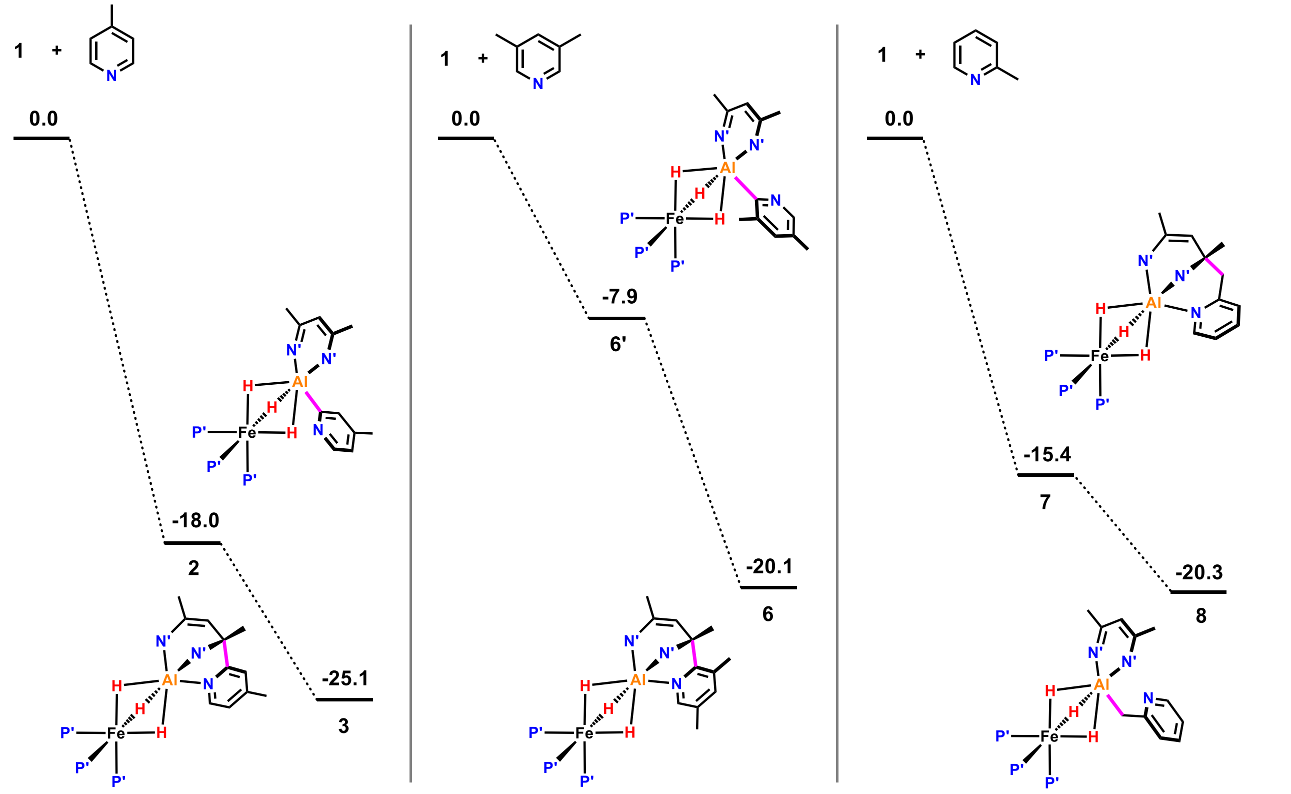

Figure S8. Calculation on the thermochemistry of the reaction of **1** with different pyridine substrates showing the energy difference between the respective C–C and C–Al bound products. P' = PMe_3, N' = N(2,4,6-MeC_6H_2). Gibbs free energies are given in kcal.mol^{-1}.

*Calculated mechanism for the reaction of **9** to **10**:*

Figure S9 shows the calculated free energy profile for the reaction of **9** to **10**. The reaction sequence is initiated by a conformational change of the pyridyl unit in **9** (**TS-1**) followed by the of the C–C bond cleavage between the β-diketiminate ligand and the activated substrate (**TS-2**). In a final step, the β-diketiminate ligand is deprotonated by the activated substrate (**TS-3**) resulting in the formation of **10** and release of 2-ethylpyridine. The overall activation energy required for this reaction is $\Delta G^{\neq}$ = 25.3 kcal mol^{-1} providing a reasonable barrier for a reaction that only occurs at elevated temperatures.

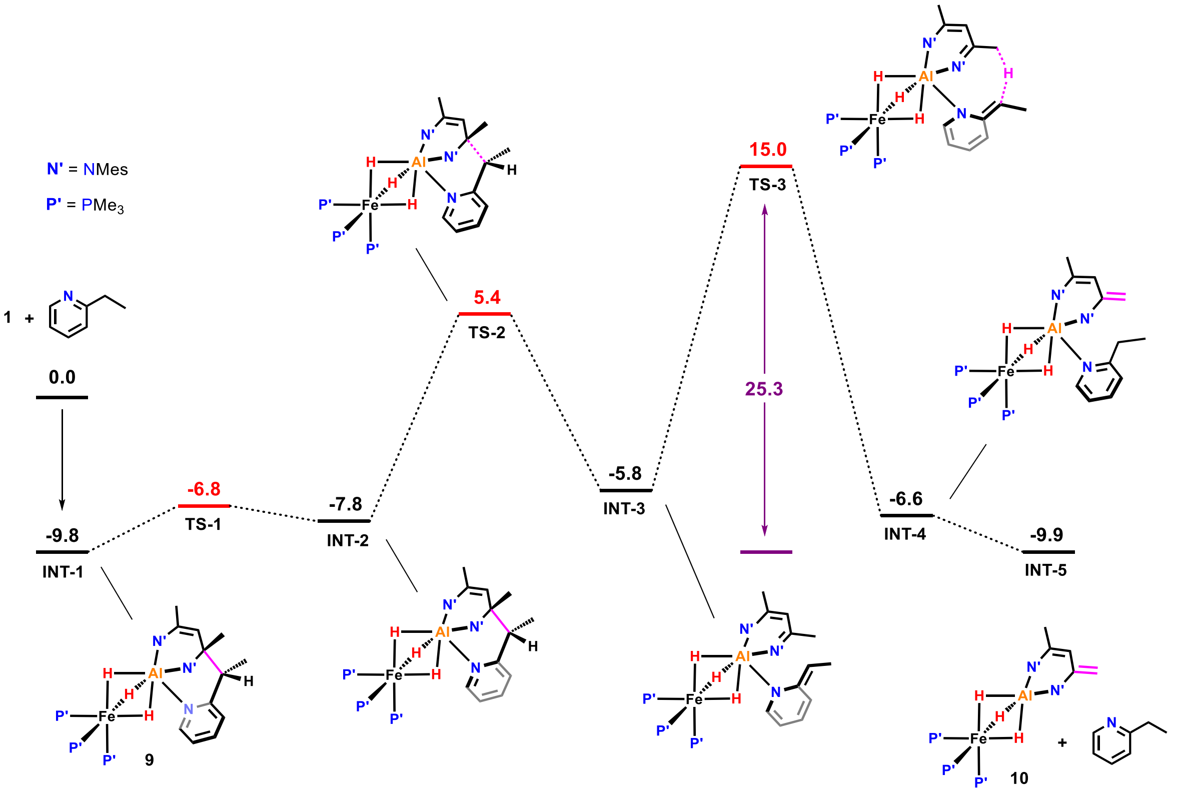

Figure S9. Calculated free energy profile for the formation of **10**. Gibbs free energies are given in kcal.mol^{-1}.

Although our calculations show that the overall reaction of **1** to **10** is exergonic (ΔG°_{298K} = -9.9 kcal mol^{-1}), the conversion of **9** to **10** under liberation of 2-ethylpyridine is almost thermoneutral (ΔG°_{298K} = -0.1 kcal mol^{-1}). However, thermal corrections have been applied taking the entropic contribution to the Gibbs free energy into account. The corrected thermodynamical data for the actual reaction conditions (80°C) in fact agree with an exergonic process (ΔG°_{353K} = -3.3 kcal mol^{-1}) for the formation of **10** (Figure S10).

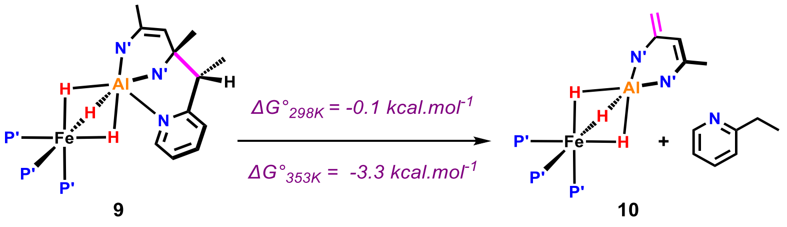

thermal correction of the Gibbs Free Energies

(freqchk utility; kcal.mol^{-1})

	298 K	353 K
1 *+ 2-ethylpyridine*	*0.0*	*0.0*
9	*-9.8*	*-6.5*
10 *+ 2-ethylpyridine*	*-9.9*	*-9.8*

Figure S10. Thermal corrections on the thermochemistry for the reaction of **9** to **10**.

5. NMR Spectra of the Isolated Compounds

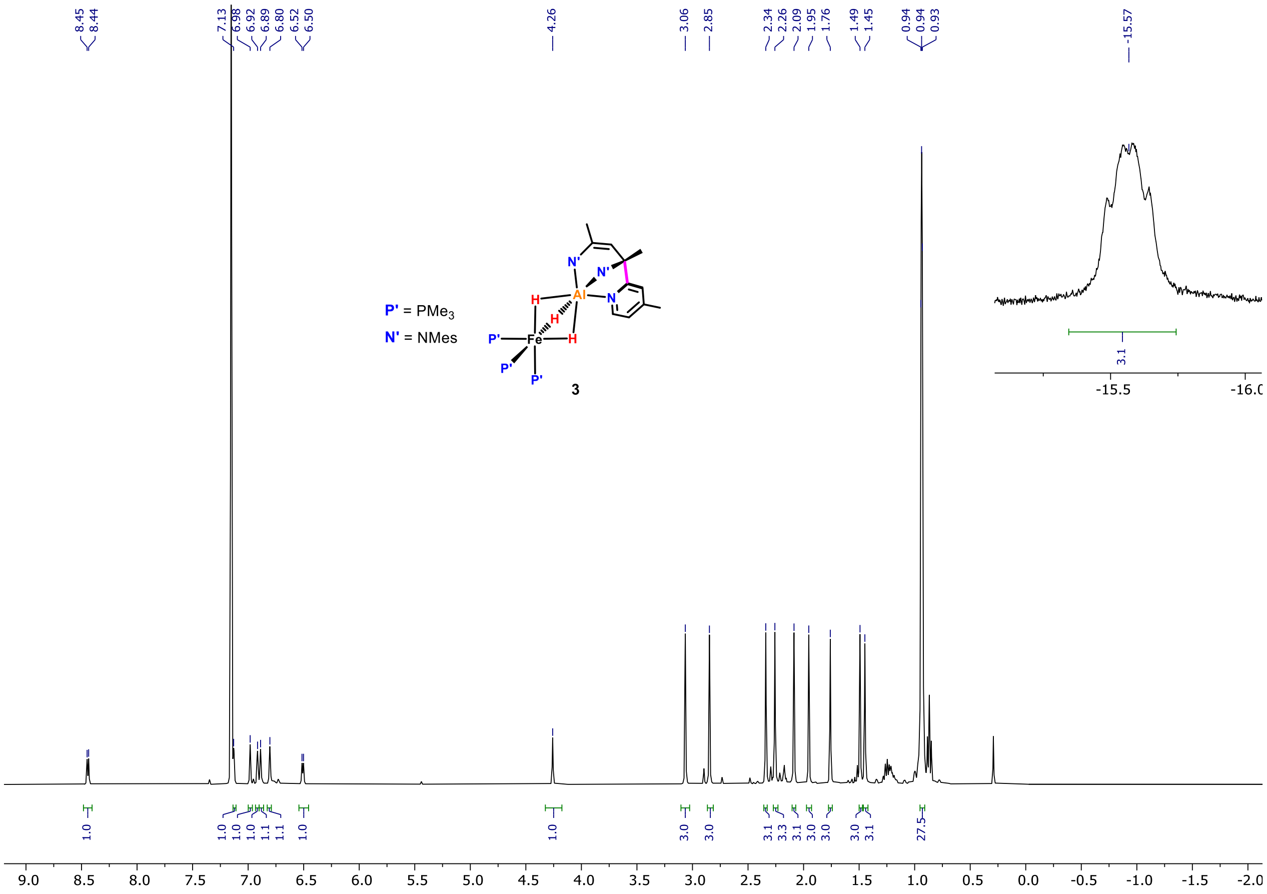

Figure S11. ^{1}H NMR of **3** (400 MHz, C_6D_6, 298 K).

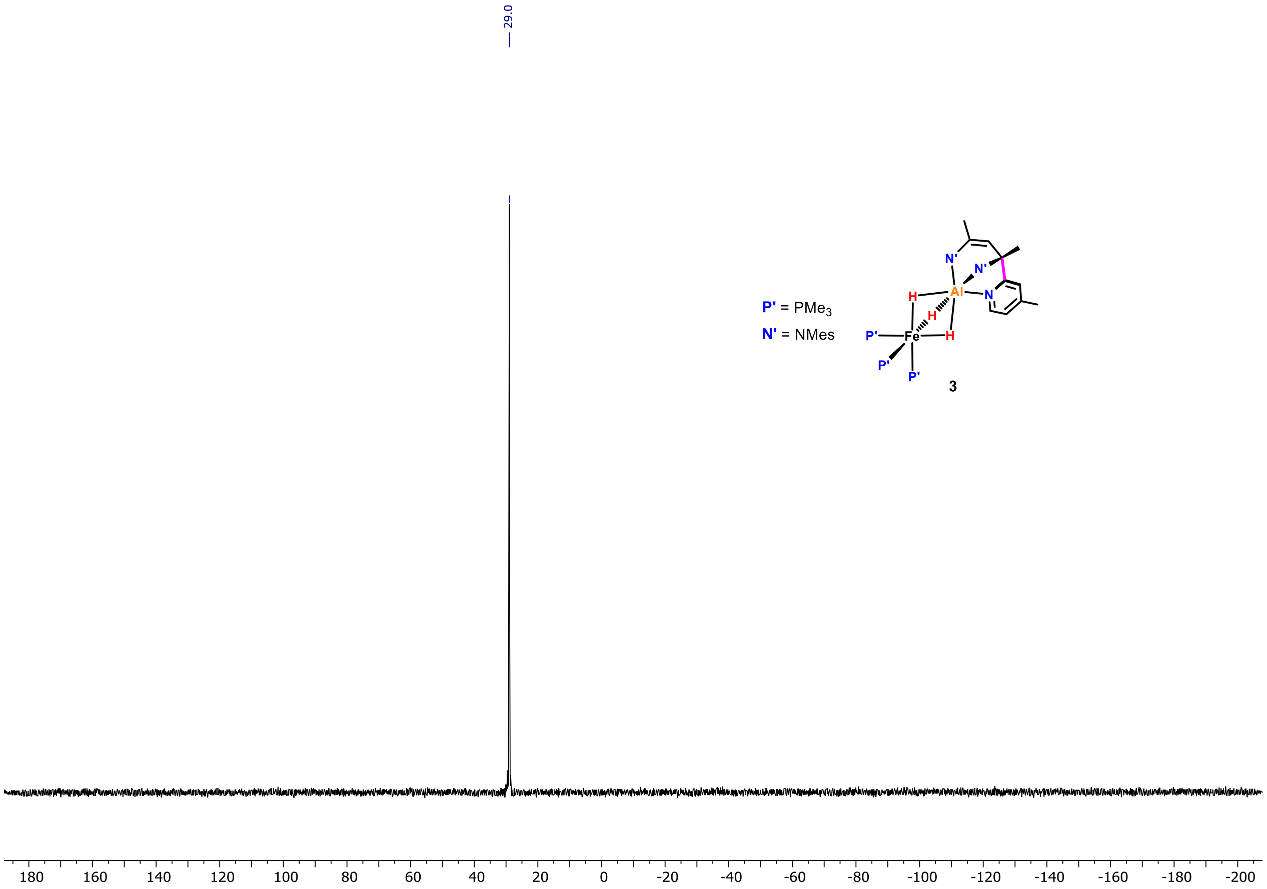

Figure S12. $^{31}P\{^{1}H\}$ NMR of **3** (162 MHz, C_6D_6, 298 K).

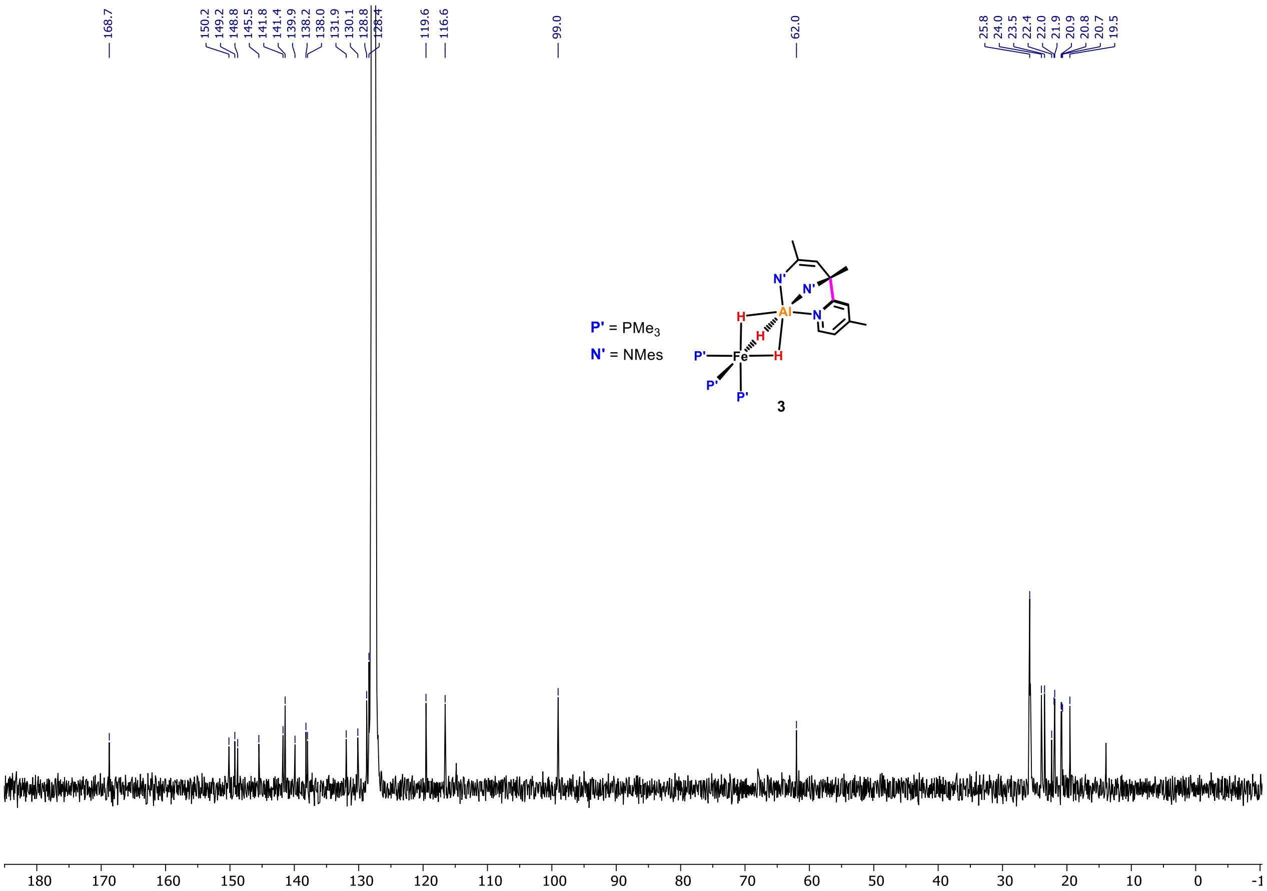

Figure S13. $^{13}C\{^{1}H\}$ NMR of **3** (101 MHz, C_6D_6, 298 K).

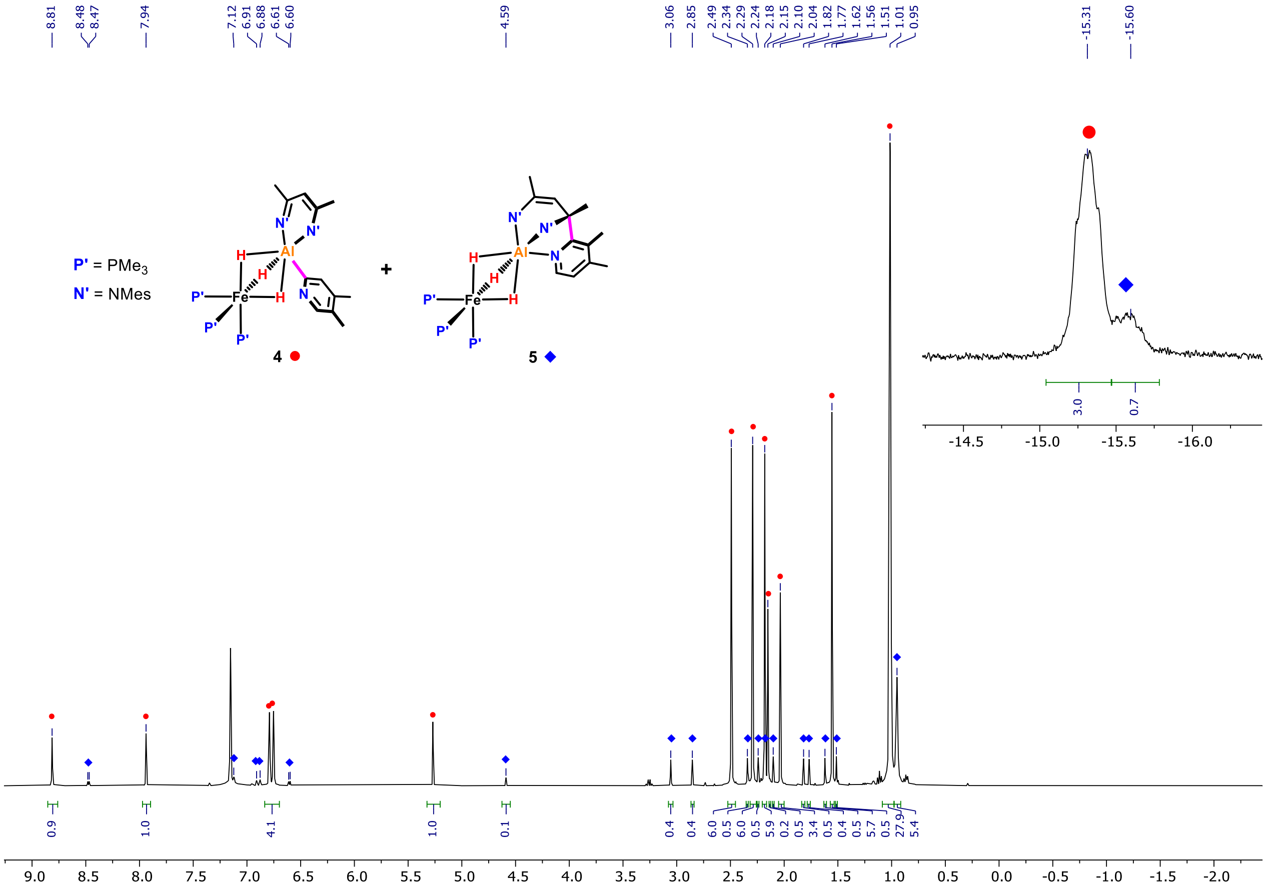

Figure S14. ^{1}H NMR (400 MHz, C_6D_6, 298 K) of **4** (red dots) and **5** (blue squares).

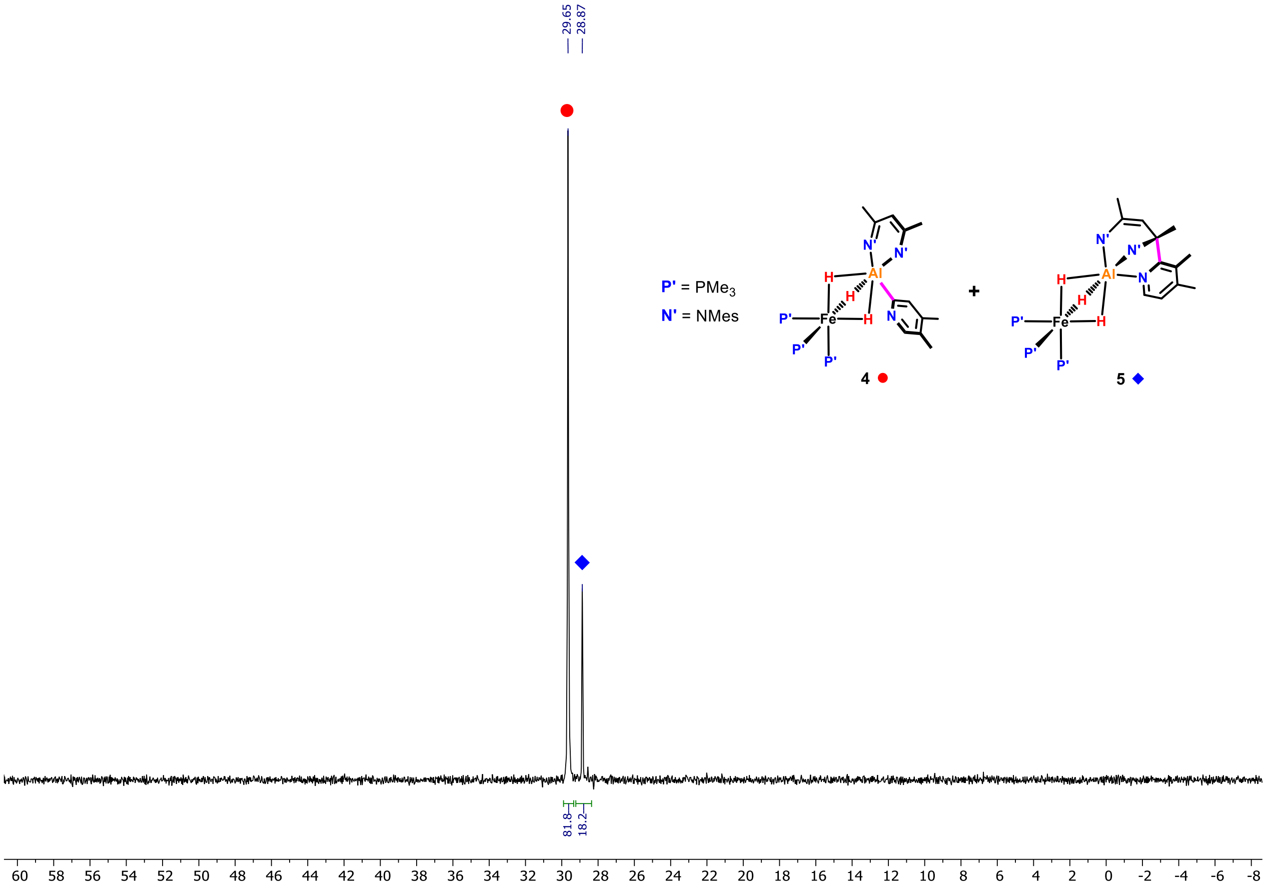

Figure S15. $^{31}P\{^{1}H\}$ (162 MHz, C_6D_6, 298 K) of **4** (red dots) and **5** (blue squares).

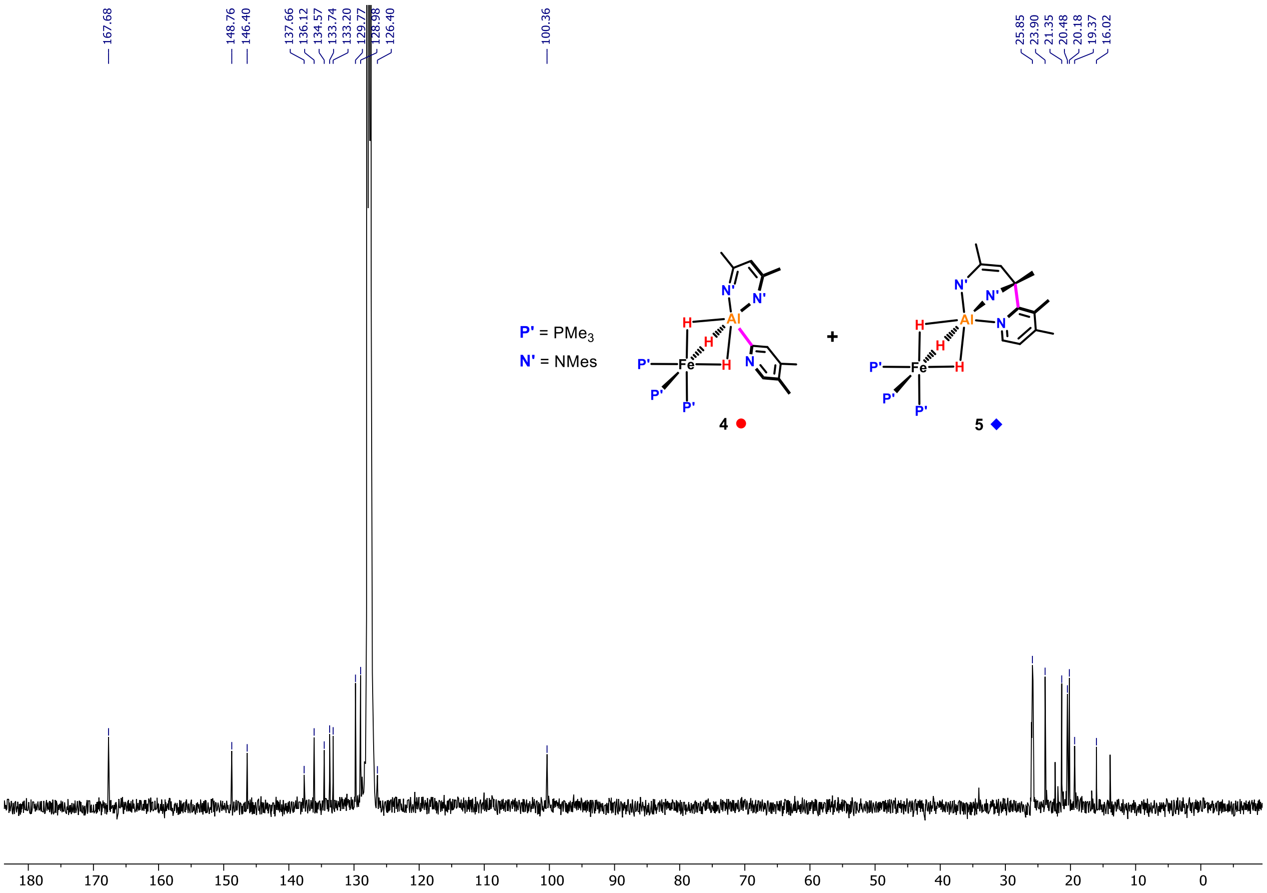

Figure S16. $^{13}C\{^{1}H\}$ NMR (101 MHz, C_6D_6, 298 K) of **4** (red dots) and **5** (blue squares).

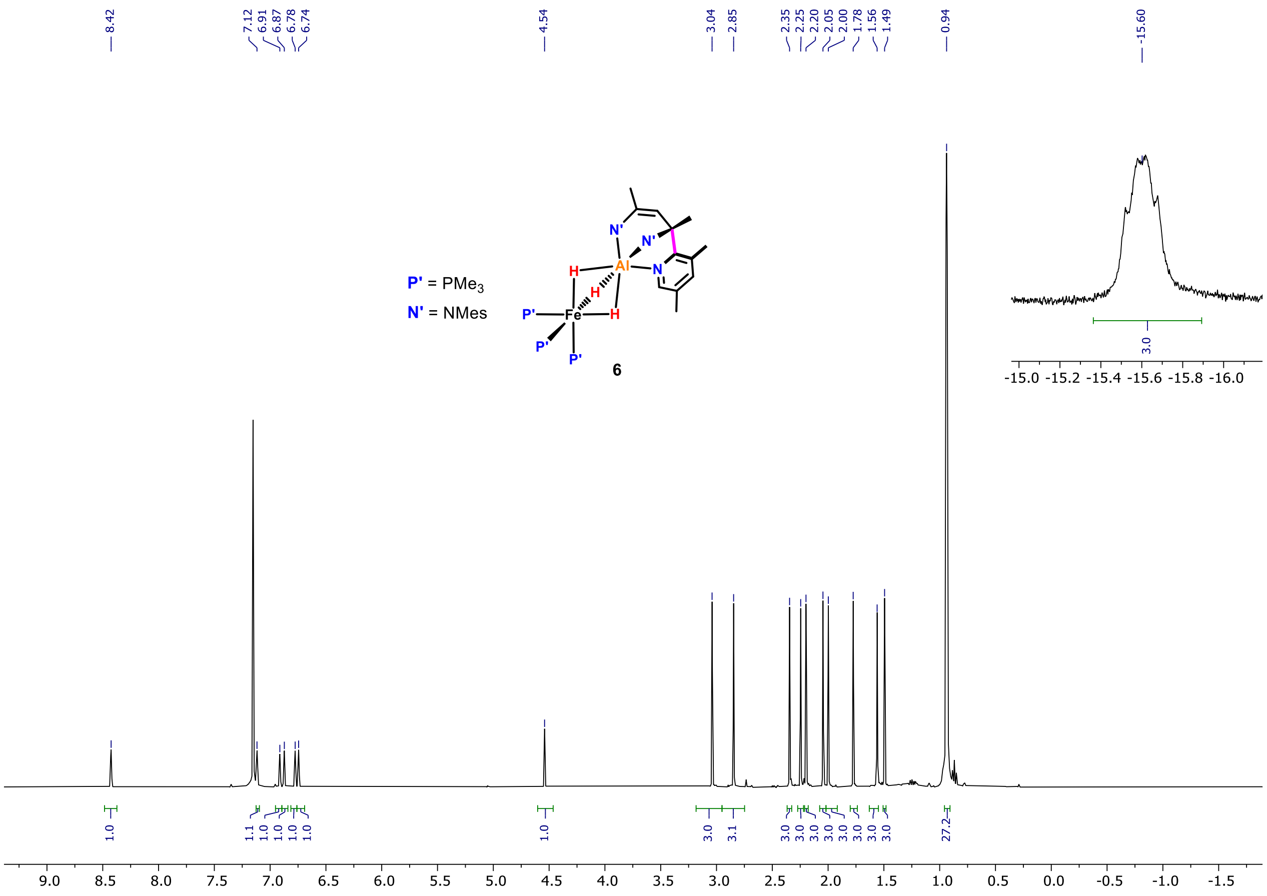

Figure S17. ^{1}H NMR of **6** (400 MHz, C_6D_6, 298 K).

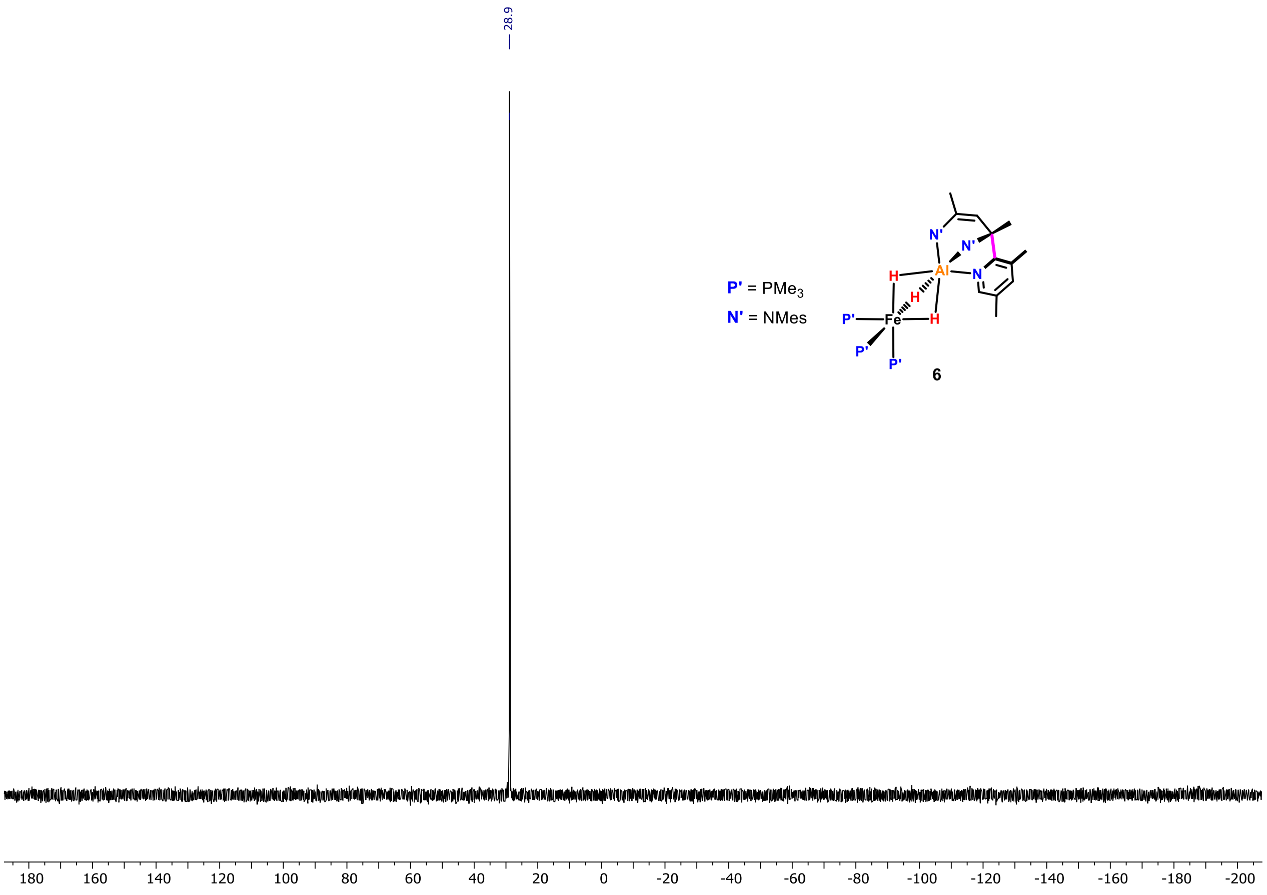

Figure S18. $^{31}P\{^{1}H\}$ NMR of **6** (162 MHz, C_6D_6, 298 K).

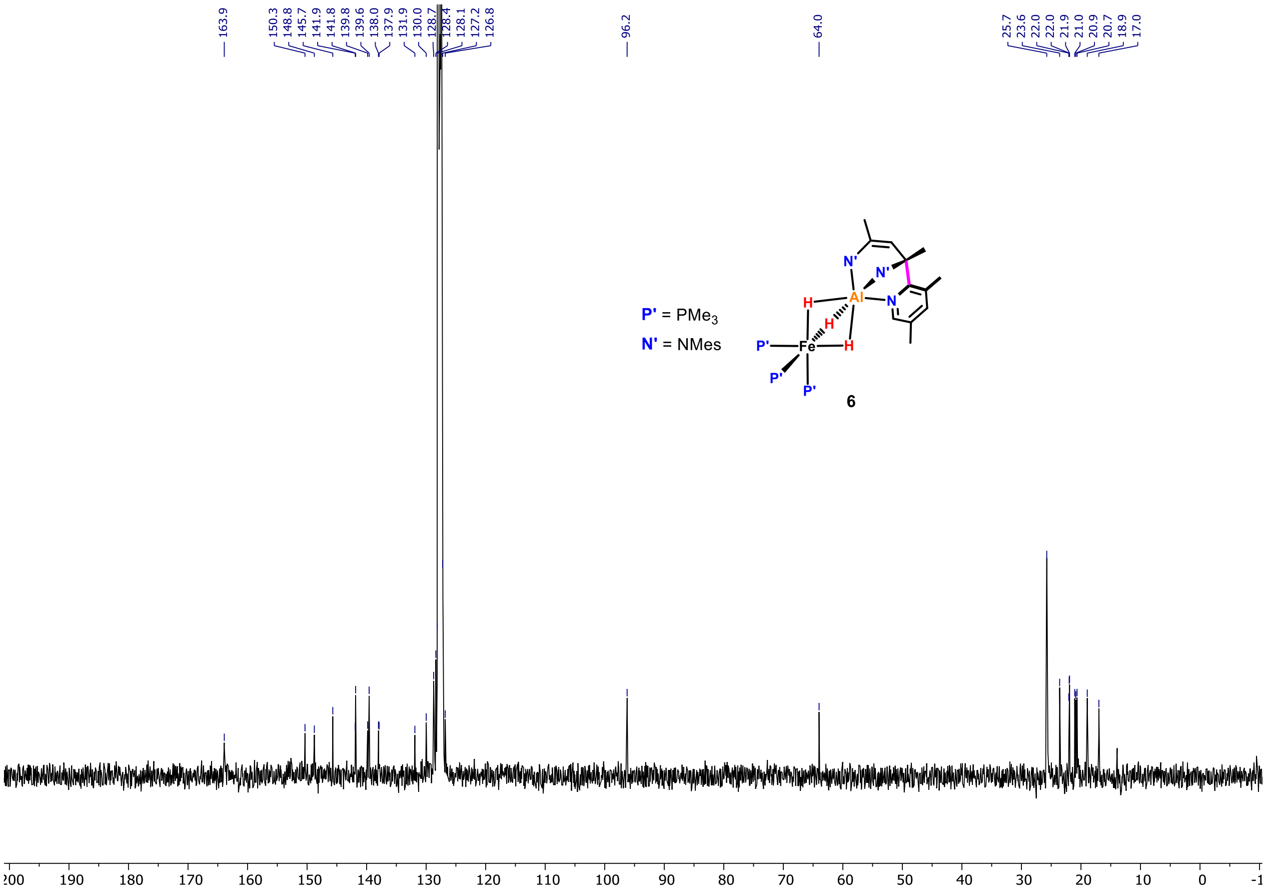

Figure S19. $^{13}C\{^{1}H\}$ NMR of **6** (101 MHz, C_6D_6, 298 K).

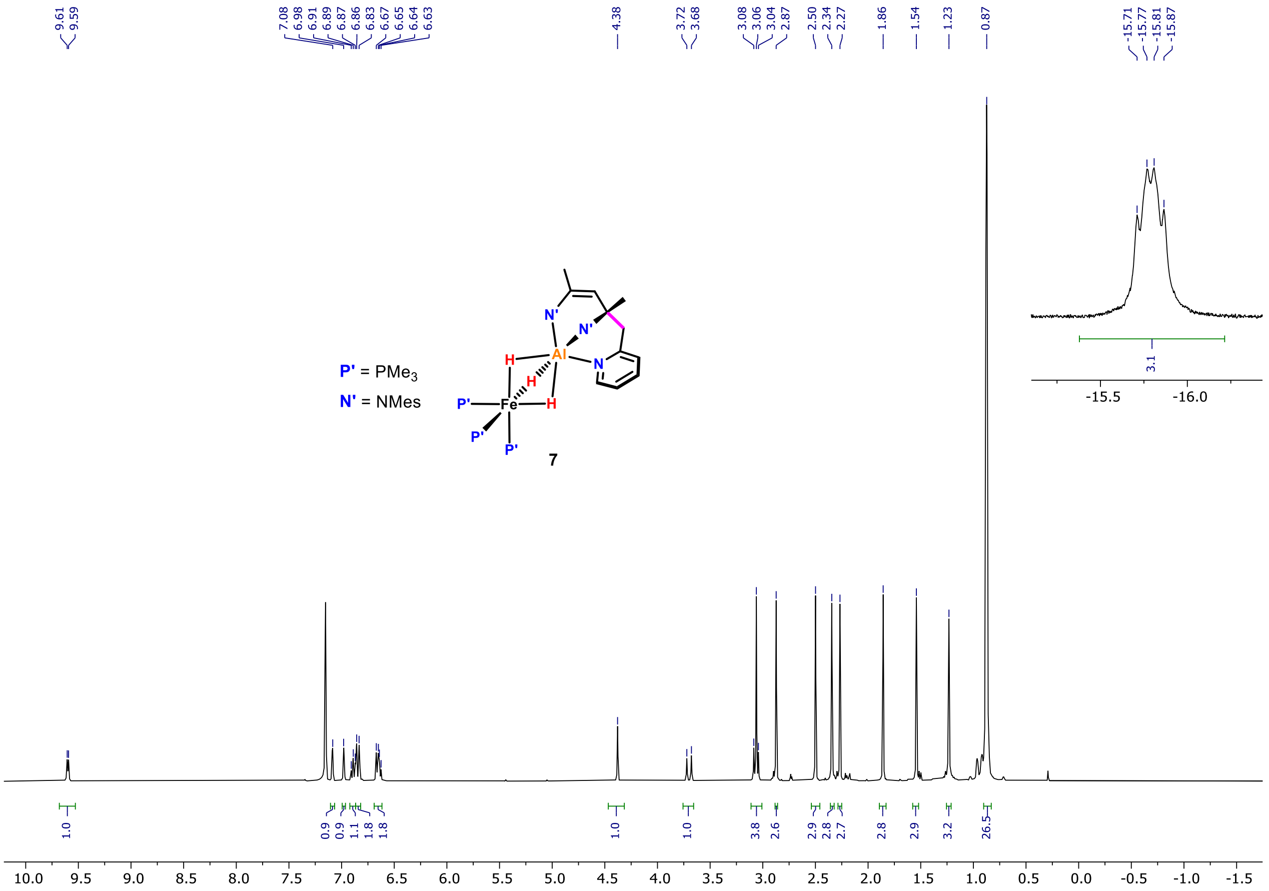

Figure S20. ^{1}H NMR of **7** (400 MHz, C_6D_6, 298 K).

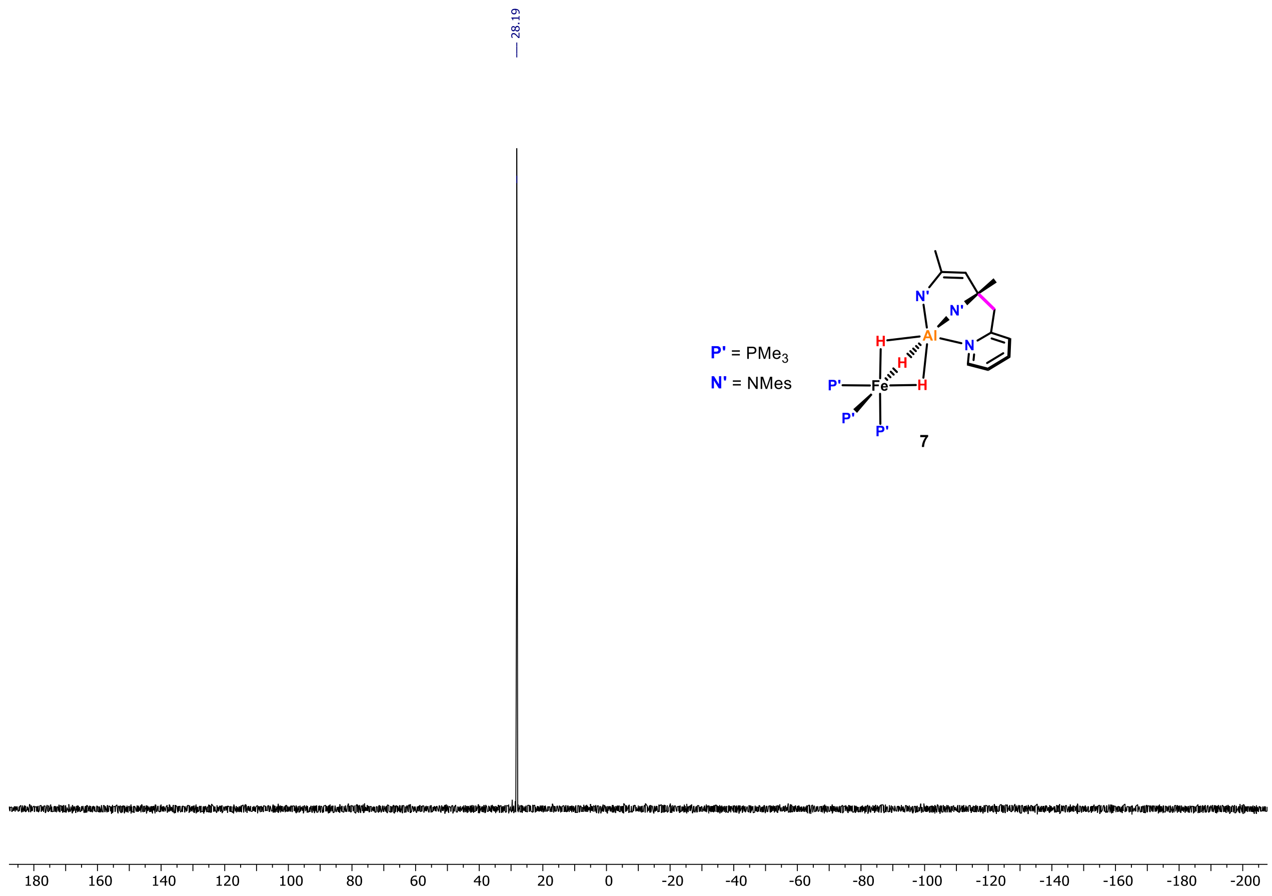

Figure S21. $^{31}P\{^{1}H\}$ NMR of **7** (162 MHz, C_6D_6, 298 K).

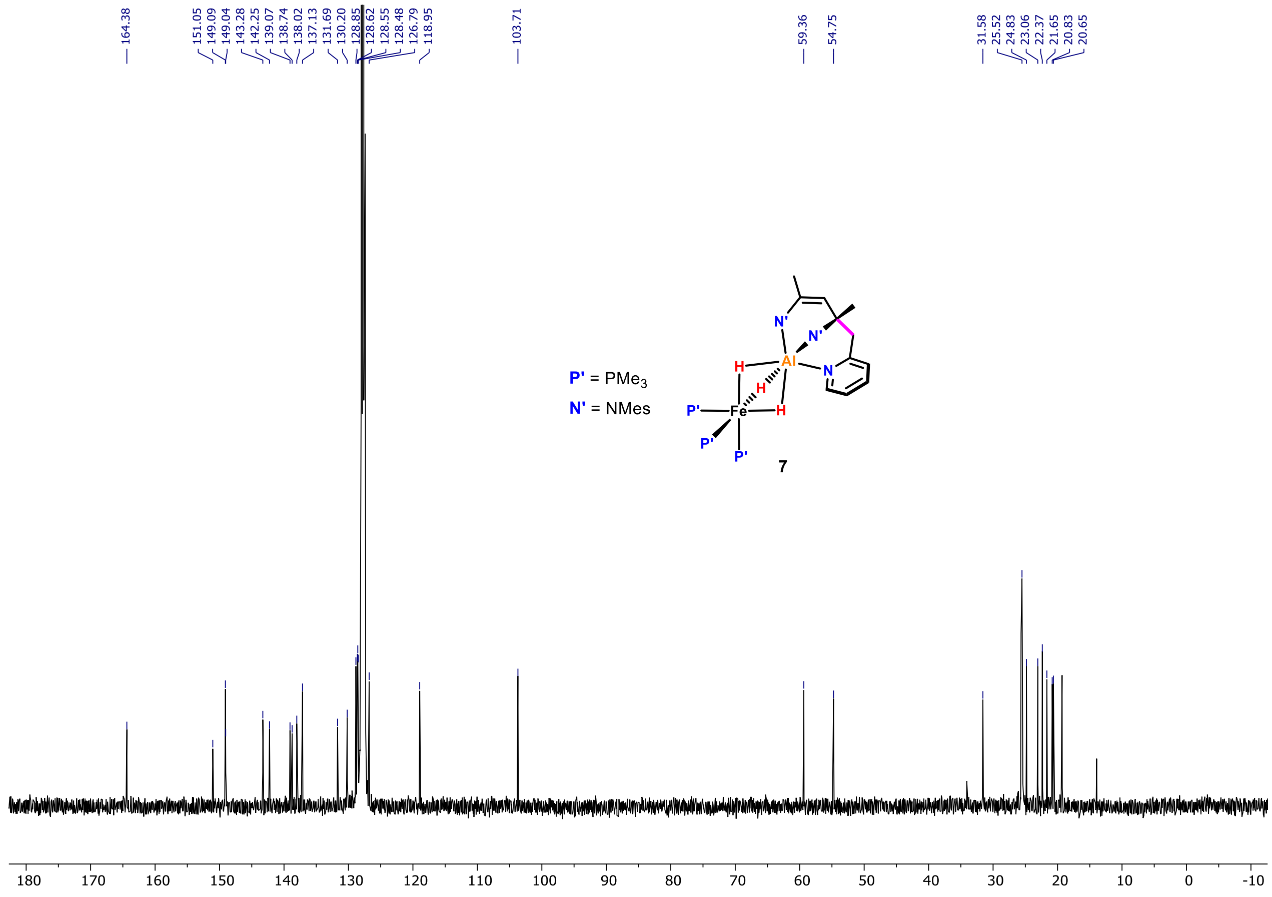

Figure S22. $^{13}C\{^{1}H\}$ NMR of **7** (101 MHz, C_6D_6, 298 K).

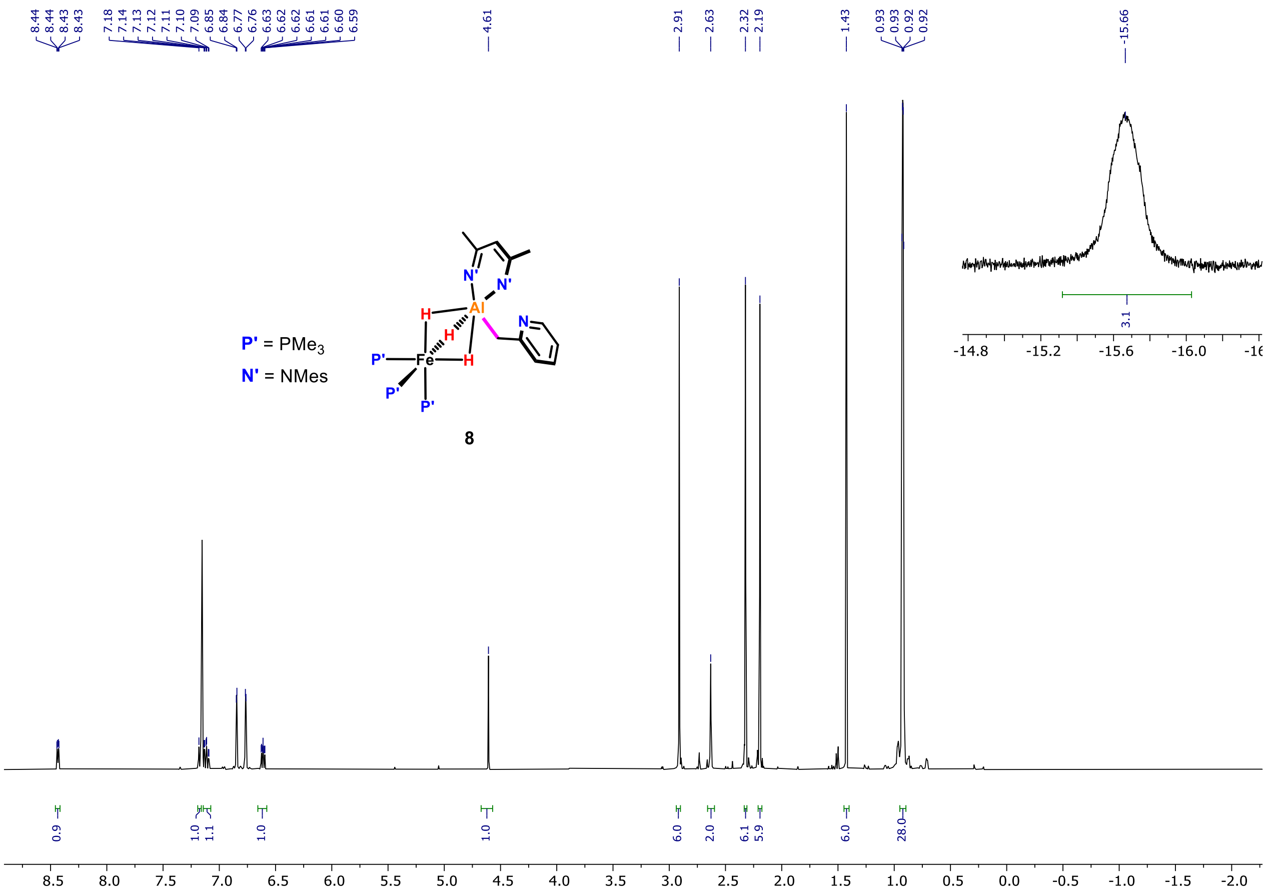

Figure S23. ^{1}H NMR of **8** (400 MHz, C_6D_6, 298 K).

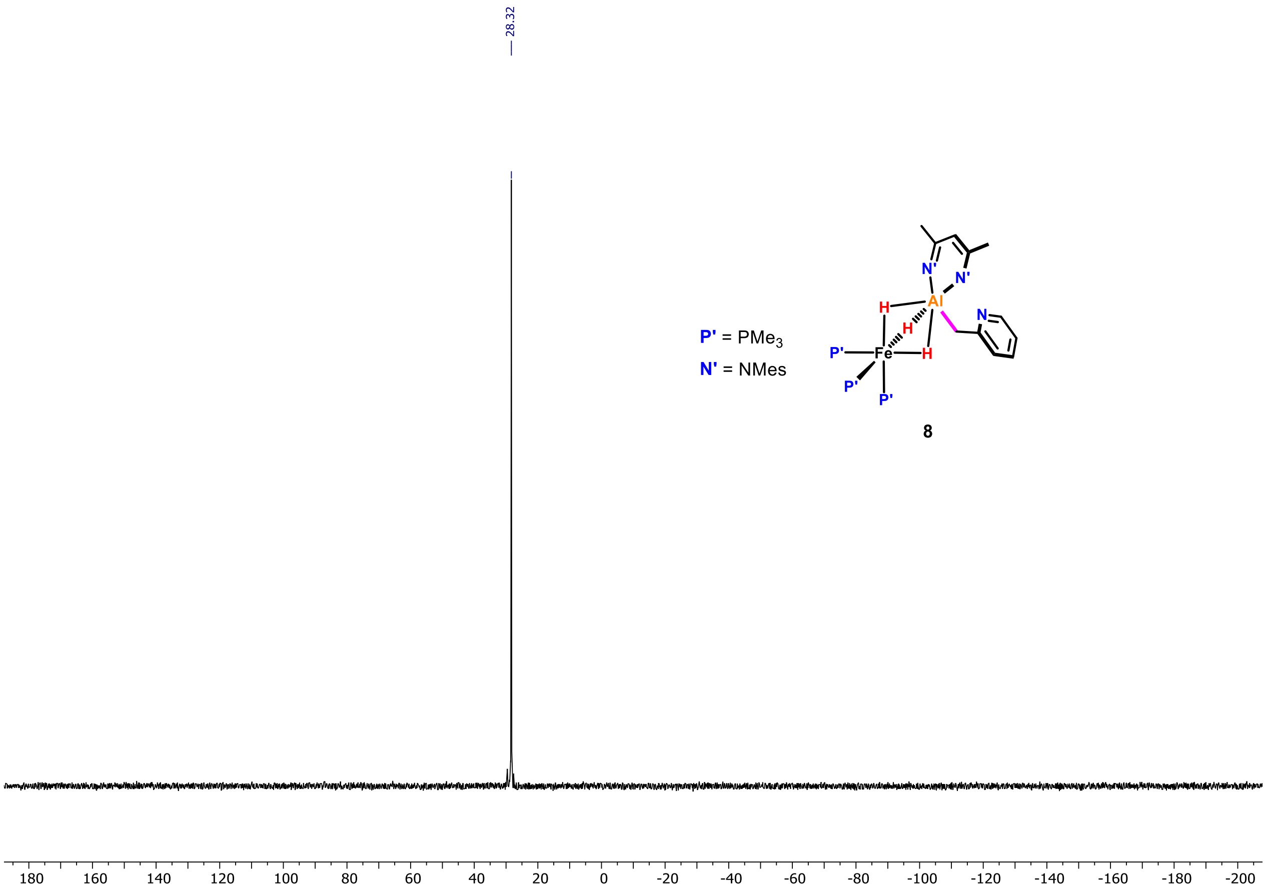

Figure S24. $^{31}P\{^{1}H\}$ NMR of **8** (162 MHz, C_6D_6, 298 K).

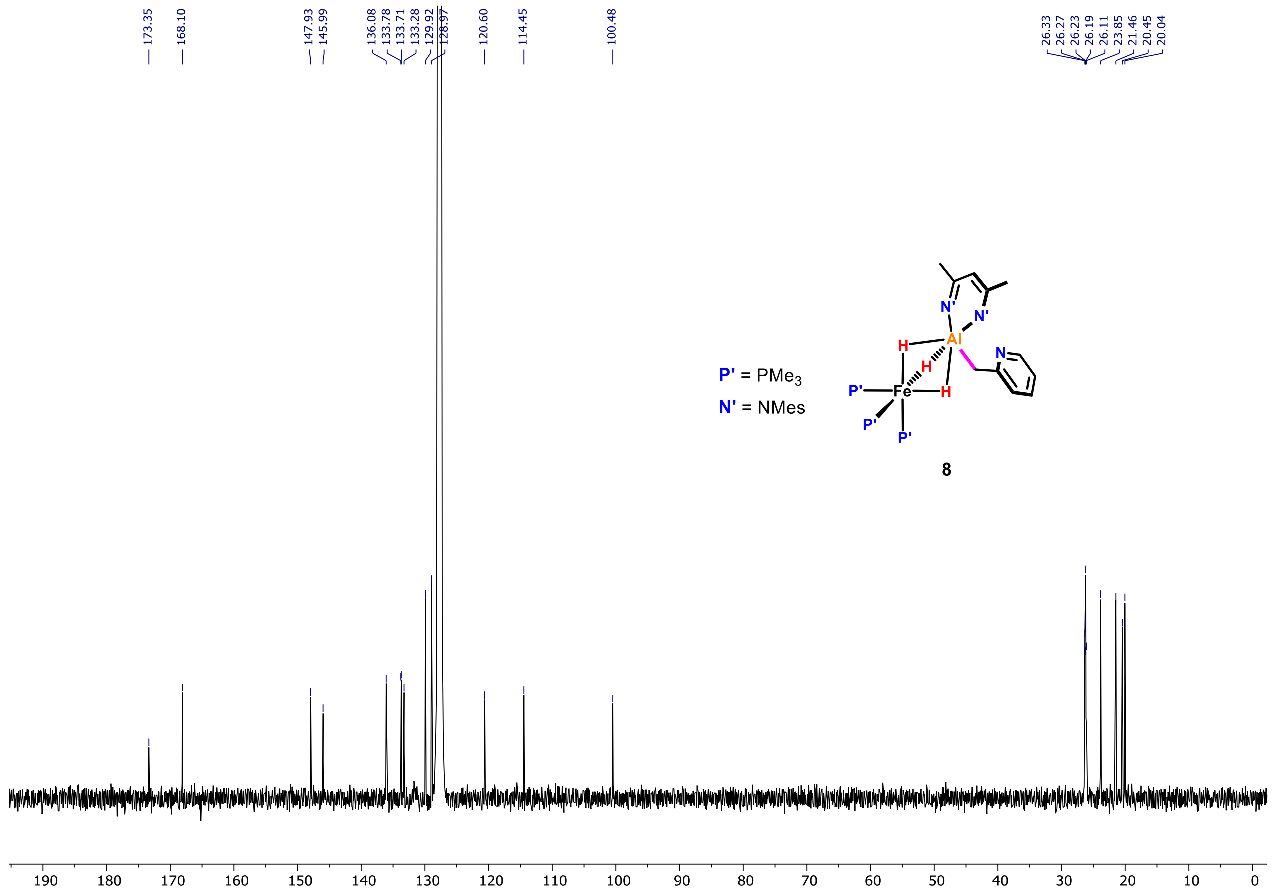

Figure S25. $^{13}C\{^{1}H\}$ NMR of **8** (101 MHz, C_6D_6, 298 K).

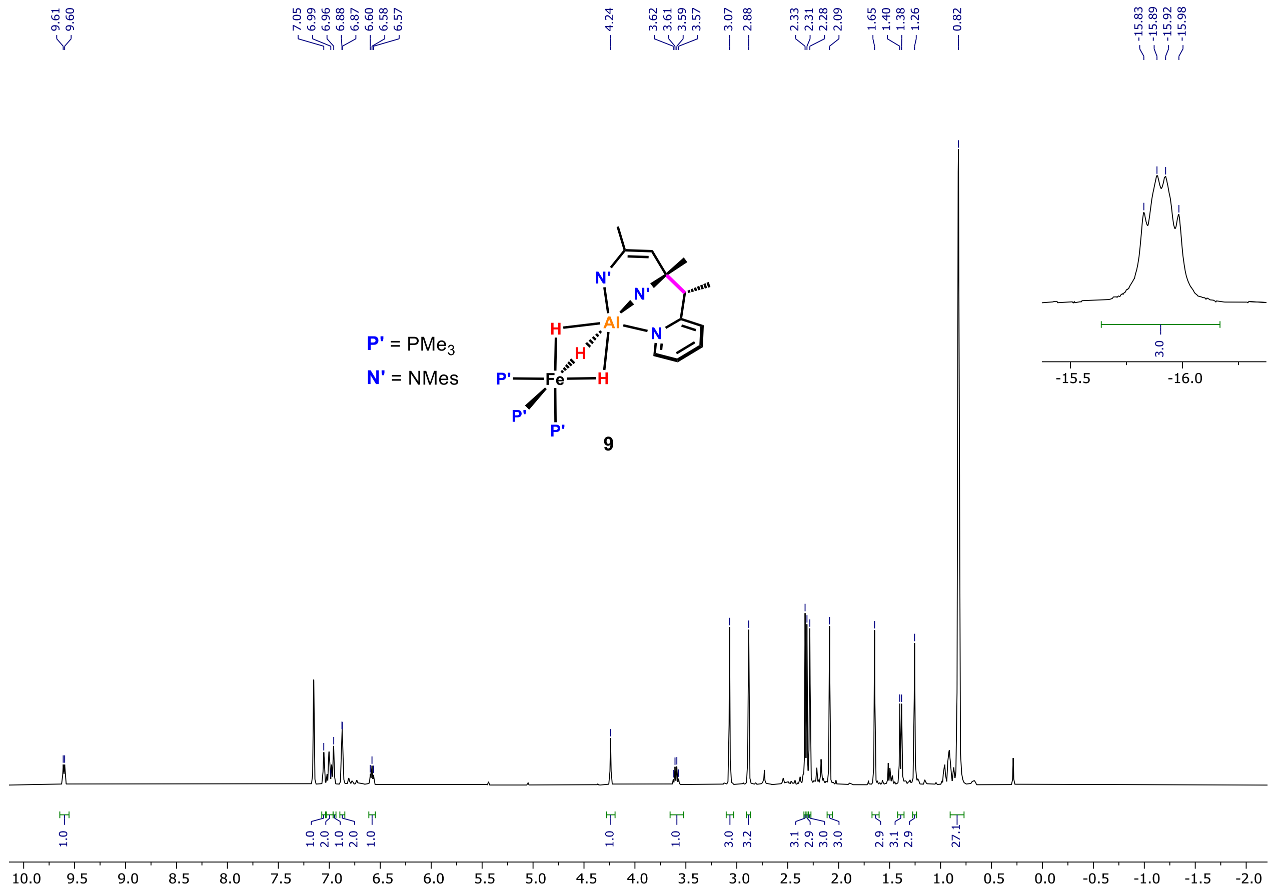

Figure S26. ^{1}H NMR of **9** (400 MHz, C_6D_6, 298 K).

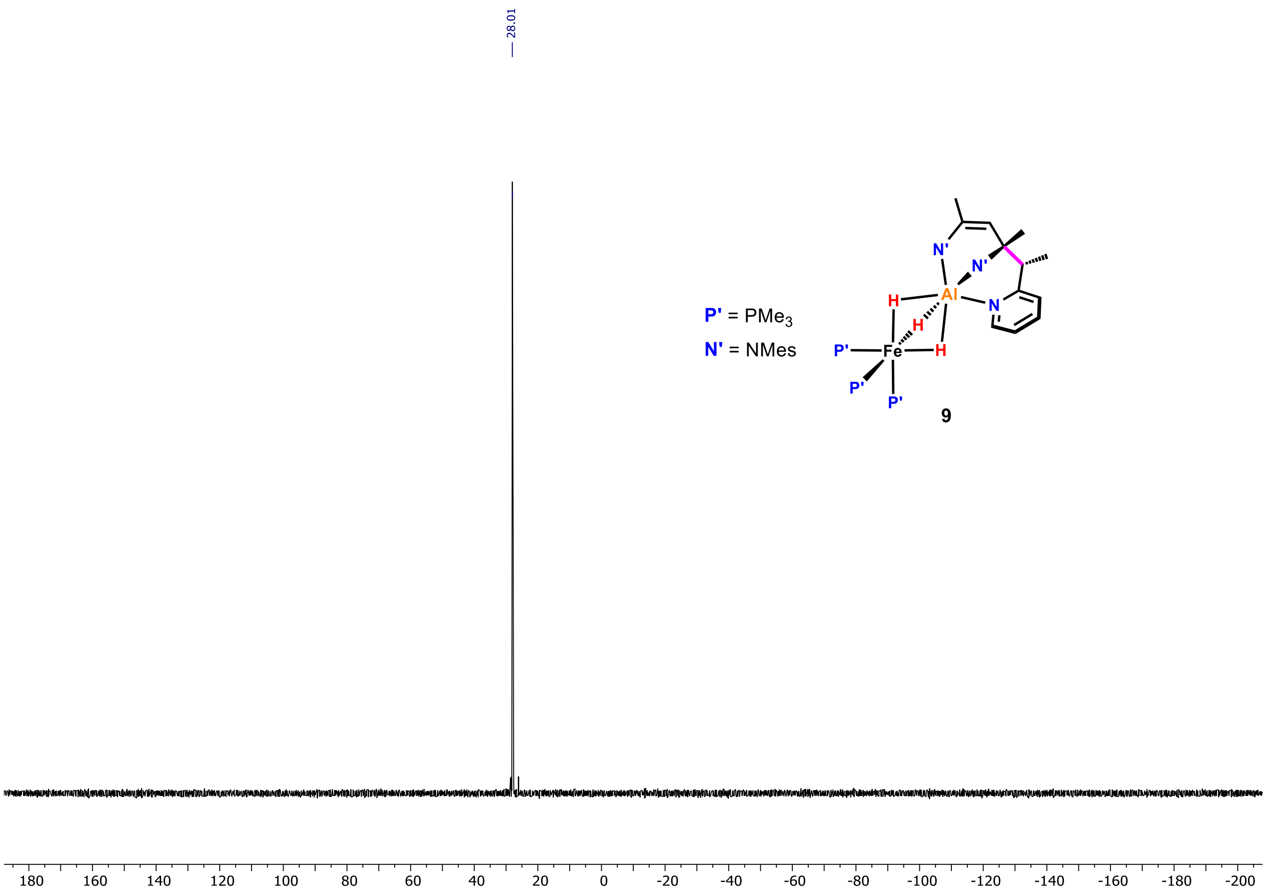

Figure S27. $^{31}P\{^{1}H\}$ NMR of **9** (162 MHz, C_6D_6, 298 K).

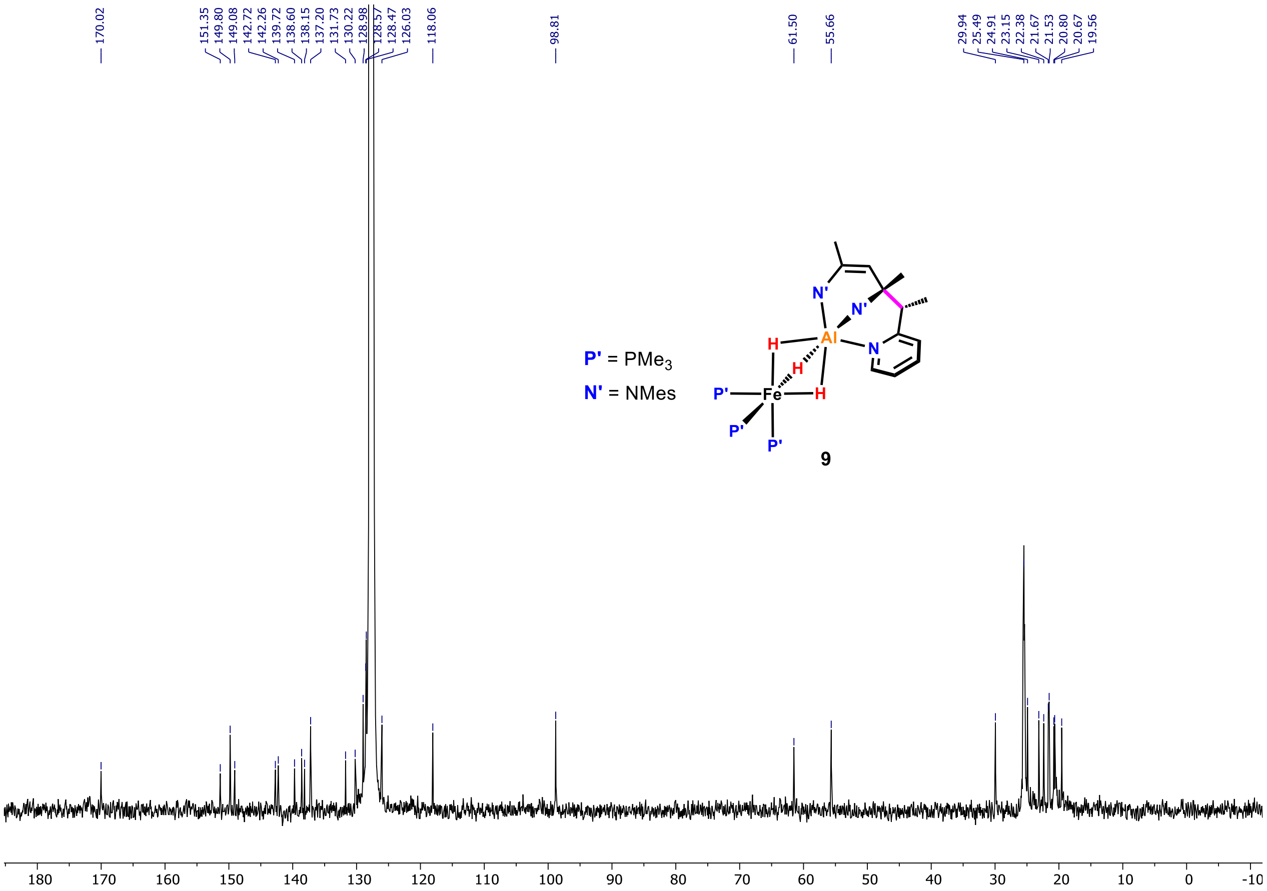

Figure S28. $^{13}C\{^{1}H\}$ NMR of **9** (101 MHz, C_6D_6, 298 K).

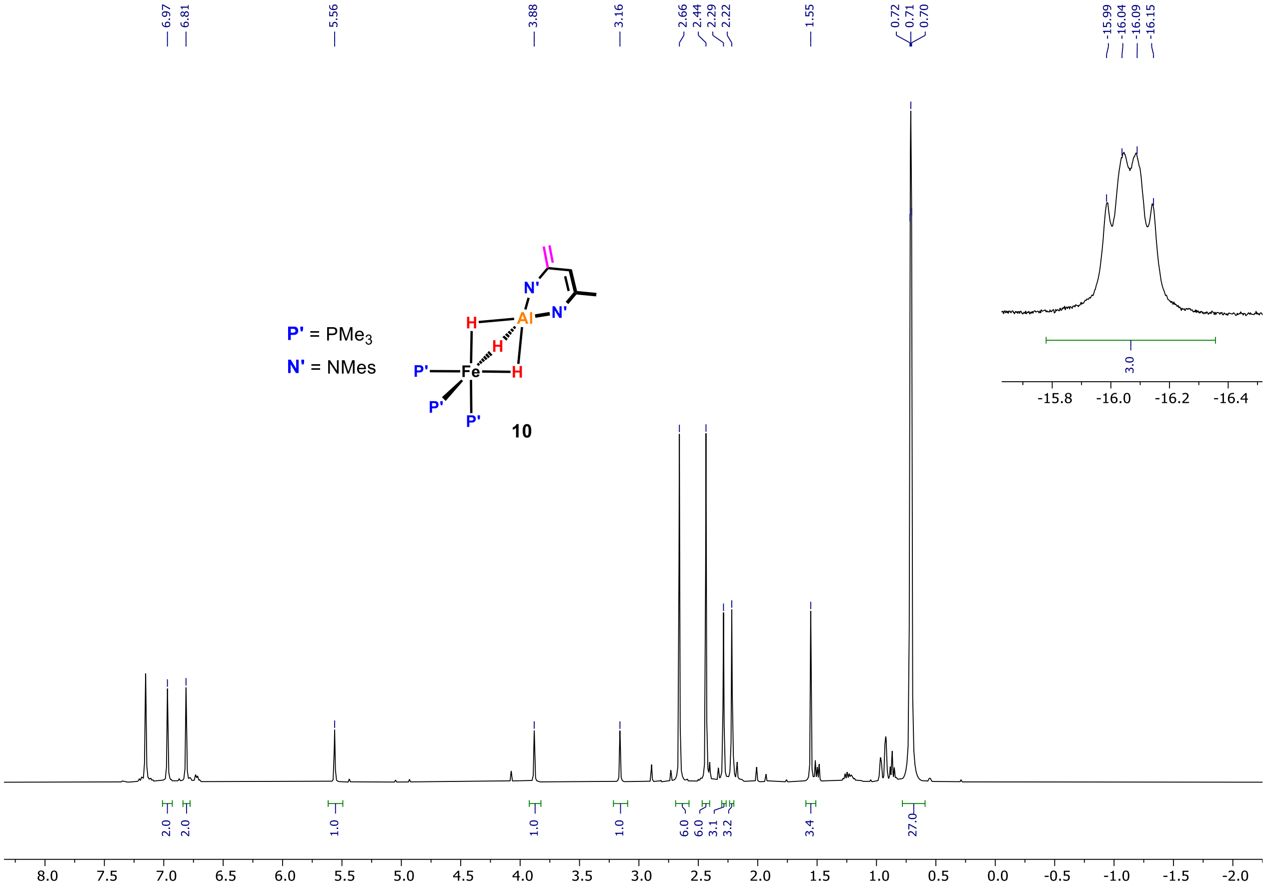

Figure S29. ^{1}H NMR of **10** (400 MHz, C_6D_6, 298 K).

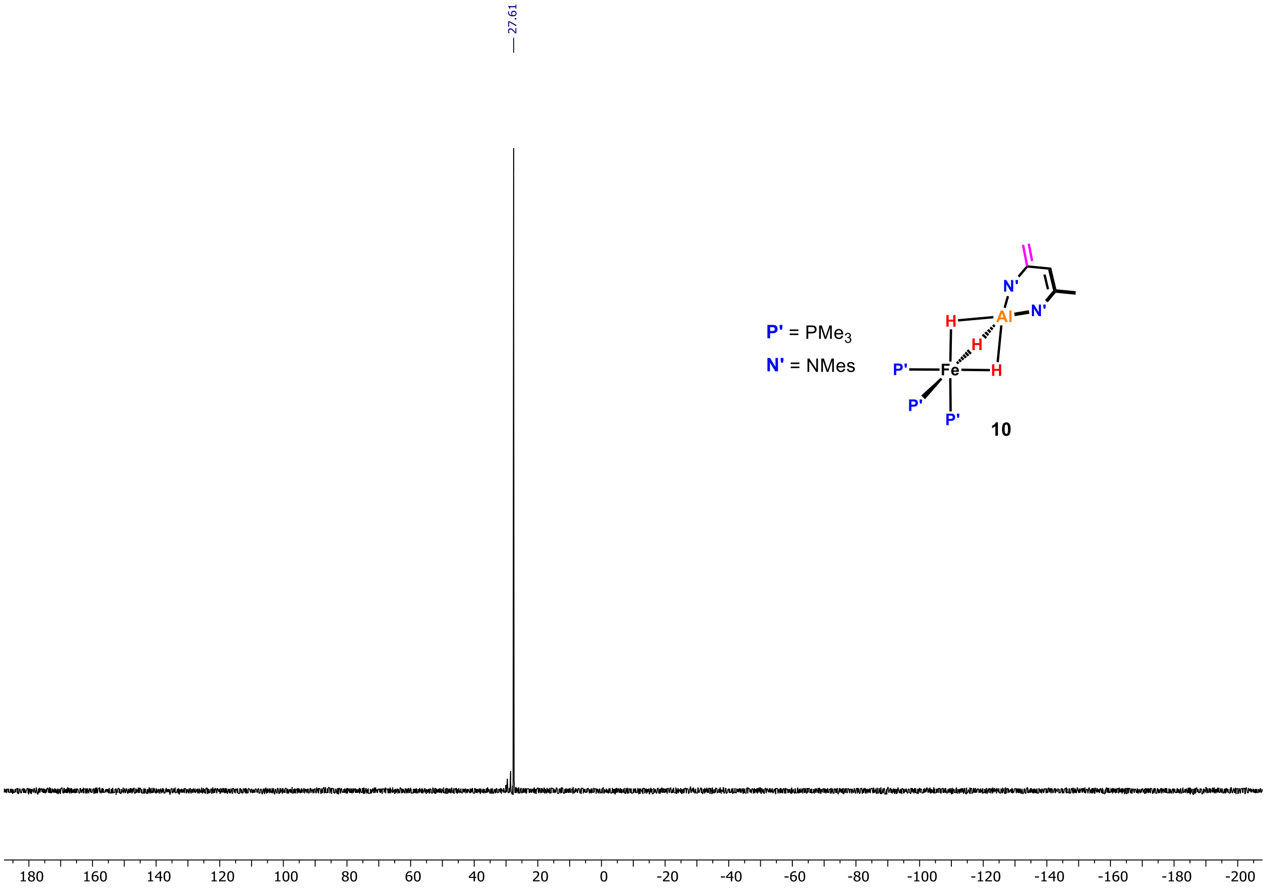

Figure S30. $^{31}P\{^{1}H\}$ NMR of **10** (162 MHz, C_6D_6, 298 K).

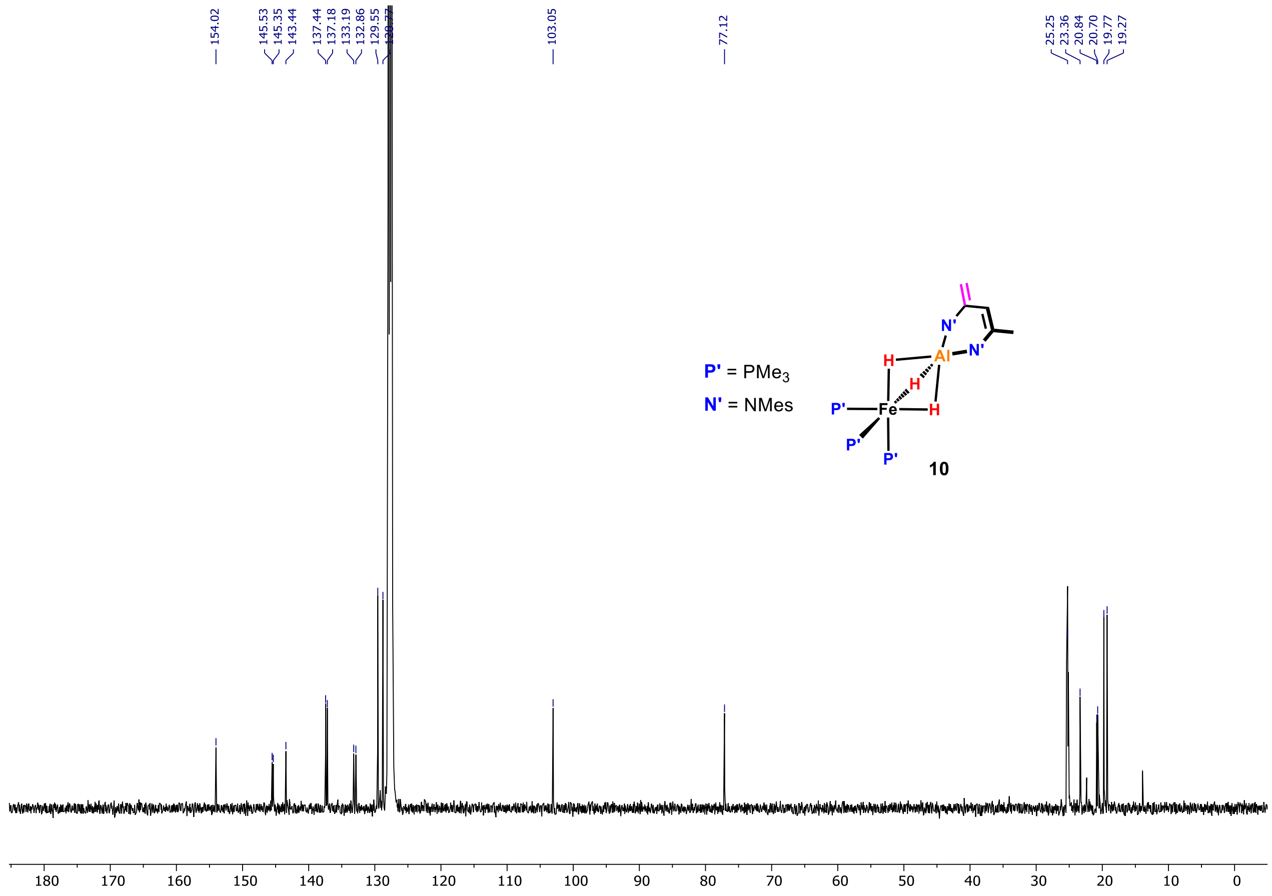

Figure S31. $^{13}C\{^{1}H\}$ NMR of **10** (101 MHz, C_6D_6, 298 K).

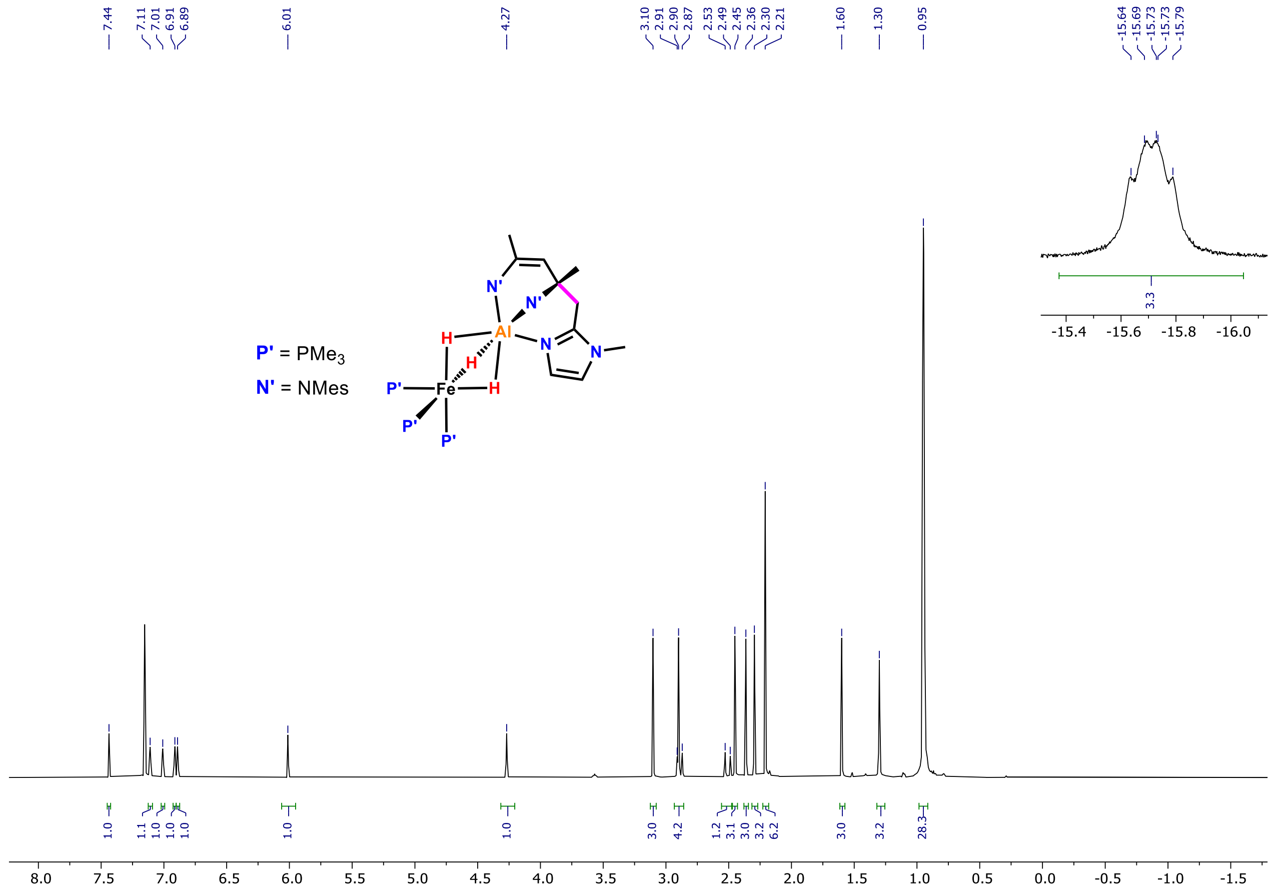

Figure S32. ^{1}H NMR of **S1** (400 MHz, C_6D_6, 298 K).

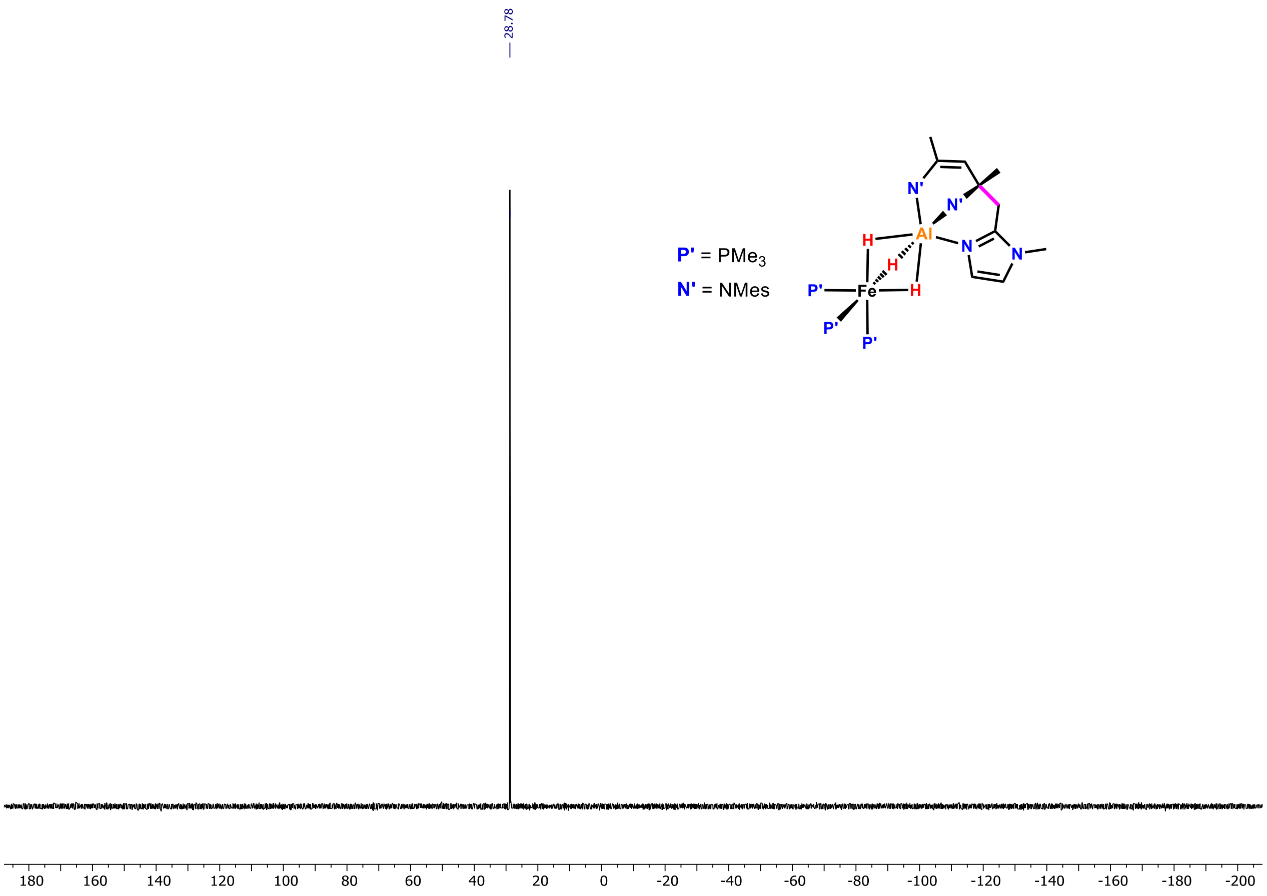

Figure S33. $^{31}P\{^{1}H\}$ NMR of **S1** (162 MHz, C_6D_6, 298 K).

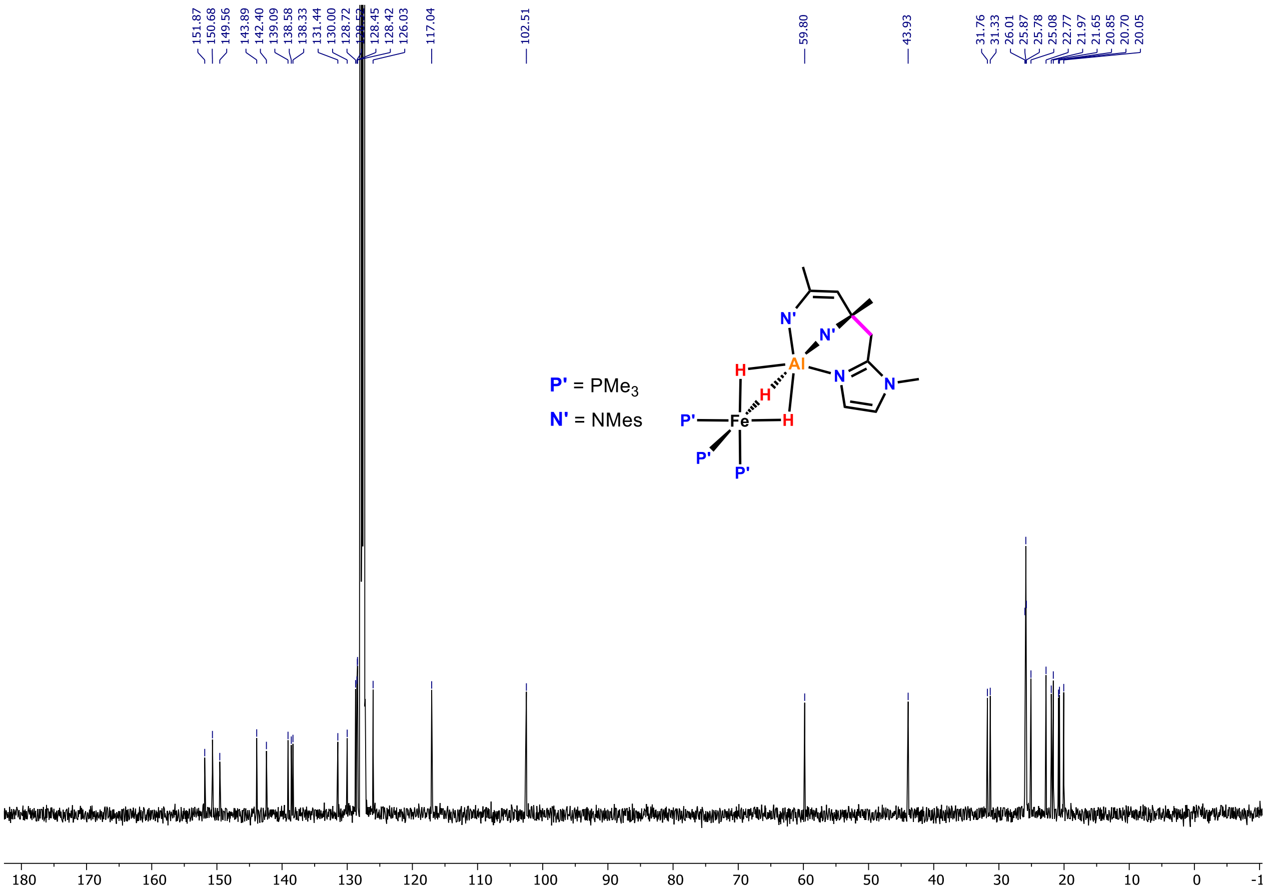

Figure S34. $^{13}C\{^{1}H\}$ NMR of **S1** (101 MHz, C_6D_6, 298 K).

6. References

[1] N. Gorgas, A. J. P. White and M. R. Crimmin, *J. Am. Chem. Soc.*, **2022**, *144*, 8770–8777.

[2] O.V. Dolomanov, L.J. Bourhis, R.J. Gildea, J.A.K. Howard, H. Puschmann, *J. Appl. Cryst.*, 2009, **42**, 339-341.

[3] Sheldrick, G. M. SHELXTL v5.1. Bruker AXS: Madison, WI 1998.

[4] Sheldrick, G. M. Crystal Structure Refinement with SHELXL. *Acta Cryst. C* **2015**, *71* (1), 3–8.

[5] Frisch, M. J.; Trucks, G. W.; Schlegel, H. B.; Scuseria, G. E.; Robb, M. A.; Cheeseman, J. R.; Scalmani, G.; Barone, V.; Mennucci, B.; Petersson, G. A.; Nakatsuji, H.; Caricato, M.; Li, X.; Hratchian, H. P.; Izmaylov, A. F.; Bloino, J.; Zheng, G.; Sonnenberg, J. L.; Hada, M.; Ehara, M.; Toyota, K.; Fukuda, R.; Hasegawa, J.; Ishida, M.; Nakajima, T.; Honda, Y.; Kitao, O.; Nakai, H.; Vreven, T.; Montgomery, J. A., Jr.; Peralta, J. E.; Ogliaro, F.; Bearpark, M.; Heyd, J. J.; Brothers, E.; Kudin, K. N.; Staroverov, V. N.; Kobayashi, R.; Normand, J.; Raghavachari, K.; Rendell, A.; Burant, J. C.; Iyengar, S. S.; Tomasi, J.; Cossi, M.; Rega, N.; Millam, J. M.; Klene, M.; Knox, J. E.; Cross, J. B.; Bakken, V.; Adamo, C.; Jaramillo, J.; Gomperts, R.; Stratmann, R. E.; Yazyev, O.; Austin, A. J.; Cammi, R.; Pomelli, C.; Ochterski, J. W.; Martin, R. L.; Morokuma, K.; Zakrzewski, V. G.; Voth, G. A.; Salvador, P.; Dannenberg, J. J.; Dapprich, S.; Daniels, A. D.; Farkas, Ö.; Foresman, J. B.; Ortiz, J. V.; Cioslowski, J.; Fox, D. J. Gaussian 09, Revision D.01; Gaussian, Inc., Wallingford, CT, **2009**.

[6] Fukui, K. The path of chemical reactions - the IRC approach. *Acc. Chem. Res.*, **1981**, *14*, 363–368.

[7] Hratchian, H. P.; Schlegel, H. B. Chapter 10 - Finding minima, transition states, and following reaction pathways on ab initio potential energy surfaces. *Theory and Applications of Computational Chemistry – The First Forty Years*, 1; Elsevier: London, 2005, 195–249.

[8] Tomasi, J.; Mennucci, B.; Cammi, R. Quantum Mechanical Continuum Solvation Models. *Chem. Rev.* **2005**, *105*, 2999–3094.

[9] Grimme, S.; Antony, J.; Ehrlich, S.; Krieg, H. A consistent and accurate ab initio parametrization of density functional dispersion correction (DFT-D) for the 94 elements H-Pu. *J. Chem. Phys.* **2010**, *132*, 154104.

[10] Head-Gordon, M.; Chai, J. D. Long-range corrected hybrid density functionals with damped atom–atom dispersion corrections. *Phys. Chem. Chem. Phys.* **2008**, *10*, 6615–6620.

[11] Hehre, W. J.; Ditchfield, R.; Pople, J. A. Self—Consistent Molecular Orbital Methods. XII. Further Extensions of Gaussian—Type Basis Sets for Use in Molecular Orbital Studies of Organic Molecules. *J. Chem. Phys.* **1972**, *56*, 2257–2261.

[12] Hariharan, P. C.; Pople, J. A. The influence of polarization functions on molecular orbital hydrogenation energies. *Theor. Chim. Acta.* **1973**, *28*, 213–222.

[13] Clark, T.; Chandrasekhar, J.; Spitznagel, G. W.; Schleyer, P. V. R. Efficient diffuse function augmented basis sets for anion calculations. III. The 3-21+G basis set for first-row elements, Li–F. *J. Comput. Chem*. **1983**, *4*, 294–301.

[14] Glendening, E. D.; Landis, C. R.; Weinhold, F. NBO 6.0: Natural Bond Orbital Analysis Program, *J. Comput. Chem.* **2013**, *34*, 1429-1437.
